# Low-Intensity Extracorporeal Shock Wave Therapy Ameliorates Detrusor Hyperactivity with Impaired Contractility via Transient Potential Vanilloid Channels: A Rat Model for Ovarian Hormone Deficiency

**DOI:** 10.3390/ijms25094927

**Published:** 2024-04-30

**Authors:** Kuang-Shun Chueh, Tai-Jui Juan, Jian-He Lu, Bin-Nan Wu, Rong-Jyh Lin, Jing-Wen Mao, Hung-Yu Lin, Shu-Mien Chuang, Chao-Yuan Chang, Mei-Chen Shen, Ting-Wei Sun, Yung-Shun Juan

**Affiliations:** 1 Graduate Institute of Clinical Medicine, College of Medicine, Kaohsiung Medical University, Kaohsiung 80708, Taiwan; space_jason@hotmail.com (K.-S.C.); chaoyuah@kmu.edu.tw (C.-Y.C.); 2 Department of Urology, Kaohsiung Municipal Ta-Tung Hospital, Kaohsiung 80661, Taiwan; 3 Department of Urology, Kaohsiung Medical University Hospital, Kaohsiung 80756, Taiwan; u9181002@gmail.com (S.-M.C.); bear5824@gmail.com (M.-C.S.); ting.wei0220@gmail.com (T.-W.S.); 4 Department of Medicine, National Defense Medical College, Taipei 11490, Taiwan; terryjuan@gmail.com (T.-J.J.); blast2337@gmail.com (J.-W.M.); 5 Emerging Compounds Research Center, Department of Environmental Science and Engineering, College of Engineering, National Pingtung University of Science and Technology, Pingtung 91201, Taiwan; toddherpuma@yahoo.com.tw; 6 Department of Pharmacology, Graduate Institute of Medicine, College of Medicine, Kaohsiung Medical University, Kaohsiung 80708, Taiwan; binnan@kmu.edu.tw; 7 Department of Parasitology, School of Medicine, College of Medicine, Kaohsiung Medical University, Kaohsiung 80708, Taiwan; rjlin@kmu.edu.tw; 8 Graduate Institute of Medicine, College of Medicine, Kaohsiung Medical University, Kaohsiung 80708, Taiwan; 9 School of Medicine, College of Medicine, I-Shou University, Kaohsiung 82445, Taiwan; ed100464@edah.org.tw; 10 Division of Urology, Department of Surgery, E-Da Cancer Hospital, Kaohsiung 82445, Taiwan; 11 Division of Urology, Department of Surgery, E-Da Hospital, Kaohsiung 824005, Taiwan; 12 Department of Anatomy, School of Medicine, College of Medicine, Kaohsiung Medical University, Kaohsiung 80708, Taiwan

**Keywords:** low-intensity extracorporeal shock wave therapy, ovarian hormone deficiency, detrusor hyperactivity with impaired contractility, transient potential vanilloid channel

## Abstract

This study explores low-intensity extracorporeal shock wave therapy (LiESWT)’s efficacy in alleviating detrusor hyperactivity with impaired contractility (DHIC) induced by ovarian hormone deficiency (OHD) in ovariectomized rats. The rats were categorized into the following four groups: sham group; OVX group, subjected to bilateral ovariectomy (OVX) for 12 months to induce OHD; OVX + SW4 group, underwent OHD for 12 months followed by 4 weeks of weekly LiESWT; and OVX + SW8 group, underwent OHD for 12 months followed by 8 weeks of weekly LiESWT. Cystometrogram studies and voiding behavior tracing were used to identify the symptoms of DHIC. Muscle strip contractility was evaluated through electrical-field, carbachol, ATP, and KCl stimulations. Western blot and immunofluorescence analyses were performed to assess the expressions of various markers related to bladder dysfunction. The OVX rats exhibited significant bladder deterioration and overactivity, alleviated by LiESWT. LiESWT modified transient receptor potential vanilloid (TRPV) channel expression, regulating calcium concentration and enhancing bladder capacity. It also elevated endoplasmic reticulum (ER) stress proteins, influencing ER-related Ca^2+^ channels and receptors to modulate detrusor muscle contractility. OHD after 12 months led to neuronal degeneration and reduced TRPV1 and TRPV4 channel activation. LiESWT demonstrated potential in enhancing angiogenic remodeling, neurogenesis, and receptor response, ameliorating DHIC via TRPV channels and cellular signaling in the OHD-induced DHIC rat model.

## 1. Introduction

A postmenopausal hypoestrogen status can induce urinary symptoms, including frequency, nocturia, urgency and urge incontinence. Up to 45% of postmenopausal women experience urogenital atrophy, detrusor hyperactivity, incontinence and recurrent urinary tract infection [1]. Abnormal detrusor activity leads to the failure of the storage function in the lower urinary tract, resulting in urinary urgency and incontinence. Detrusor hyperactivity with impaired contractility (DHIC) has been identified as a contributor to lower urinary tract symptoms (LUTS) among the elderly population [2,3]. Abarbanel and Marcus [4] showed that the prevalence of DHIC among elderly patients reached up to 18%. In a video urodynamic study, Ong and Kuo [5] documented that among the female patients enrolled with stress urinary incontinence (SUI), 19.4% exhibited detrusor overactivity (DO), while 8.4% displayed detrusor underactivity (DU). Although DHIC was associated with urgency urinary incontinence (UUI), it did not significantly contribute to the underactive bladder (UAB) population [6]. In a clinical context, DHIC has been characterized by DO symptoms during the filling phase and underactive detrusor contractions during the voiding phase, indicating a weak detrusor [7]. However, among the elderly, DHIC entails pathological traits linked to both UAB and overactive bladder (OAB) [8]. DHIC is identified through urodynamic diagnosis, marked by involuntary detrusor contractions or reduced compliance during filling, a voiding pattern of low pressure and low flow, and urinary retention, as observed in urodynamic studies. However, some studies have also suggested an enhanced bladder sensation and a normal detrusor pressure pattern in patients with DHIC [9]. The treatments for DHIC include lifestyle modification, pelvic floor muscle training, pharmacotherapy with antimuscarinic and/or β3-adrenergic receptor agonists, and sacral neuromodulation surgery. β3-Adrenergic receptor agonists, which can relax the bladder detrusor muscle without inhibiting the effect of acetylcholine during detrusor contraction, have also been used in DHIC treatment to improve urgency and voiding efficiency [10]. Moreover, intravesical onabotulinumtoxin A injection has the potential to improve the urgency symptom for UUI in DHIC [11]. The concurrent administration of intravesical onabotulinumtoxin A injection and solifenacin could alleviate the DO symptom and extend the time between subsequent reinjections [12]. In addition, sacral neuromodulation has the potential to improve UUI and address compromised detrusor contractile function. Hennessey and Hoag documented that sacral neuromodulation in patients with DHIC exhibited the ability to alleviate symptoms of DO and reduce PVR volume [13]. However, the pathophysiology and mechanism of DHIC is not clearly defined. Additional investigations are required to establish substantial evidence for the efficacy and safety of treatments for DHIC and improve patient quality of life.

Ovariectomized rats exhibited voiding dysfunction, including increased postvoiding residual urine (PVR), reduced voiding efficiency, and detrusor hyperactivity, as well as altered coordination between the bladder detrusor and urethral sphincter [14]. In female estrogen receptor β^−/−^ mice, the pathological morphologies of urothelial ulceration, atrophy and bladder hyperactivity were shown to be compatible with interstitial cystitis and bladder pain syndrome (IC/BPS) in human [15]. In our earlier study, we showed that the bilateral ovariectomy (OVX)-induced OHD rats reduced bladder compliance and elevated levels of oxidative damage, interstitial fibrosis, and apoptosis of bladder mucosa [16]. The OVX-treated rabbits exhibited significant vascular degeneration and a decrease in vascular density. Nevertheless, treatment with estradiol induced angiogenic remodeling and an increase in vascular density within the detrusor smooth muscle bundles to ameliorate the symptoms of bladder overactivity [17,18]. Although bladder pathophysiological changes in OVX animals have published, the mechanism of bladder dysfunction is still vague. In rat, both estrogen receptors α and β are found within bladder afferent neurons located in the lumbosacral dorsal root ganglia [19]. Both of these receptors are present in the same neurons that are also costained with transient receptor potential vanilloid 1 (TRPV1), a nociceptive ion channel sensitive to capsaicin [20]. The effect of 17-estradiol is to activate estrogen-receptor signaling and inhibit the activation of TRPV1 by capsaicin in rat nociceptor neurons, which can modulate bladder pain [21]. Therefore, estrogen might reduce the excitatory effects of capsaicin and modulate pain to influence voiding function.

The therapeutic potential of intravesical TRPV1 agonists (capsaicin and resiniferatoxin (RTX)) have been widely applied to study OAB/DO, IC/BPS, neurogenic and idiopathic DO (NDO and IDO), and bladder outlet obstruction (BOO)-related OAB/DO [22]. Some medicines for TRP channels, including TRPV1, TRPV4, transient receptor potential ankyrin type 1 (TRPA1), and transient receptor potential melastatin type 8 (TRPM8), can ameliorate OAB symptoms [22,23]. Several members of the TRP channel superfamily are found to express in the lower urinary tract, including not only neuronal fibers, but also urothelial, suburothelial, and muscular layers [24]. In clinical trials, the overexpression of TRPV1 [25] and P2X3 receptors [26] in the urothelium was shown in DO patients with OAB symptoms. Zhang et al. found that the expression of TRPV1 in the urothelium of female OAB patients was meaningfully higher than in healthy patients [27], and RTX, as well as capsaicin, could block TRPV1, modulate P2X, and decrease OAB symptoms [28]. Previous studies also revealed that repeated stimulation of capsaicin and RTX on TRPV1 would cause a refractory desensitized state and a decrease in sensory symptoms of OAB [28]. Clinically, patients with NDO and IDO treated with RTX and BTX-A exhibited increases in the volume at the onset of the first detrusor contraction and bladder capacity. Moreover, this treatment approach led to improvements in urinary incontinence symptoms and overall quality of life [29]. In rats, intravesical instillation of 30 μM capsaicin was found to induce DO, increase micturition pressures, and decrease bladder capacity [30]. Additionally, the TRPV4 channel is thought to play a role in the mechanosensory pathway, potentially influencing the release of sensory mediators like ATP through the modulation of afferent nerve activity triggered by bladder filling [24,31]. In cystitis rats, the administration of HC-067047, a selective TRPV4 antagonist, resulted in an augmentation of functional bladder capacity and a decrease in micturition frequency [32]. In rats with DO accompanied by BOO, the expression of TRPV4 in the bladder was elevated [33]. Moreover, Deruyver et al. found that the application of the TRPV4 agonist GSK1016790A through intravesical administration led to an elevation in voiding frequency and a decrease in PVR in a rat model featuring DU induced by pelvic nerve injury [34].

In the lower urinary tract, TRPV channels/receptors are mainly involved in nociception and mechanosensory transduction, which play important roles in regulating detrusor contractility and urothelial barrier function [35]. TRPV1 and TRPV4, Ca^2+^-permeable channels, are expressed in not only neuronal afferent fiber but also urothelial, suburothelial, and muscular layers [24,36,37]. The influx of Ca^2+^ through different TRP channels induces cell depolarization in afferent nerve fibers and initiates Ca^2+^-dependent signaling responses. The capsaicin (vanilloid) receptor TRPV acts as a mediator of extracellular Ca^2+^ influx in response to the depletion of intracellular Ca^2+^ stores [38]. The role of TRPV1 is required for urothelial functional properties, including the release of nitric oxide and increase in intracellular Ca^2+^ after vanilloid application [39]. In addition, the role of TRPV4 in the urothelial layer is associated with the adherent junction and implicated in the regulation of urothelial permeability. The calcium-permeable TRPV4 channel is also present on detrusor smooth muscles to modulate detrusor activity. In rat urothelial cells, the activation of TRPV4 induces intracellular Ca^2+^, leading to ATP release [40]. Moreover, the elevation of TRPV4 levels is related to Ca^2+^ concentration and atrial muscle hyperactivity [41]. The role of the TRPV channel involved in intracellular Ca^2+^ through the calcium channel for the activation of the detrusor smooth muscle is still controversial, since the modification of the TRPV might be a key point to treat detrusor hyperactivity.

Interstitial cells (ICs) in bladder tissues, including interstitial Cajal cells (ICCs) and interstitial Cajal-like cells (ICLCs), can participate in modulating neurotransmission at nerve endings and smooth muscles, which regulate bladder activity by regulating the Ca^2+^ concentration [42,43,44]. One study discovered that ICCs from the small intestine of mice exhibit spontaneous Cl^−^ currents activated by Ca^2+^ [45]. Furthermore, some studies have documented the involvement of both high-conductance Cl^−^ channels and inwardly rectifying Cl^−^ channels in the pacemaker activity of ICCs [46,47]. Recently, it has been demonstrated that bladder ICs in humans, guinea pigs, and pigs express TRPA1 [48]. In a study by Zhao et al., it was discovered that human suburothelial ICs express functional TRPA1, TRPV2, TRPV4, and Piezo1 channels, and they release ATP upon activation of these channels [49].

Calcium release from the endoplasmic reticulum (ER)/sarcoplasmic reticulum (SR) is mediated by two families of calcium channels, as follows: the ryanodine receptor (RyR) and the inositol trisphosphate receptor (IP3R) [50,51]. RyRs in the detrusor muscle can activate calcium-activated potassium and chloride channels. This leads to the generation of spontaneous transient outward and inward currents. A positive feedback mechanism facilitates calcium oscillation, achieved by integrating a reliance on the SR calcium concentration within the SERCA model [52].

In human clinical trials, low-intensity extracorporeal shock wave therapy (LiESWT) has been extensively applied in the treatment of different urological diseases, including OAB [53], SUI [54], IC/BPS, chronic prostatitis/chronic pelvic pain syndrome (CP/CPPS) [55,56,57,58,59], and erectile dysfunction (ED) [60,61,62,63,64]. In the treatment of refractory IC/BPS, a 4-week LiESWT (2000 shocks administered weekly at a frequency of 3 Hz and an energy level of 0.25 mJ/mm^2^) to the suprapubic bladder area demonstrated improvements in bladder frequency, reductions in inflammation and pain, and a notable decrease in the VAS for Pain scale [65]. The therapeutic efficacy of 8 weeks of LIESWT (0.25 mJ/mm^2^, 3000 pulses and 3 Hz) in 58 female postmenopausal participants with OAB was also found to promote bladder regeneration, ameliorate OAB symptoms, and improve the urodynamic parameters, including voided urine volume, maximum urinary flow rate (Qmax), postvoid residual urine (PVR) volume, and functional bladder capacity [53,66]. Furthermore, multiple randomized, placebo-controlled trials have substantiated that LiESWT markedly enhances pain relief and life quality and ameliorates voiding dysfunction in patients with CP/CPPS compared to placebo interventions [55,67,68]. Moreover, in a rat model, Wang et al. reported that LiESWT (0.02 mJ/mm^2^ and 400 pulses) for 4 weeks applied to streptozotocin-induced diabetic rats resulted in improvements in diabetic bladder dysfunction and urinary incontinence. These studies revealed that LiESWT ameliorates bladder wall composition, enhances bladder and urethra muscle contractile functions, increases bladder nerve innervation, activates bladder muscle regeneration, and promotes urethra continence [69]. Our prior findings demonstrated that the therapeutic effectiveness of LiESWT led to enhanced voided volume and mitigation of OAB symptoms induced by OHD in a rat model. The potential mechanism of LiESWT involves the modulation of peripheral and central sensitization as a means of treating CP/CPPS in capsaicin-induced prostatitis [70]. Research was also conducted on a neural model that explores the relationship between chronic pain, pain relief facilitated by LiESWT, and the mechanism of pain inhibition [71]. However, LiESWT application may result in the selective loss of sensory unmyelinated nerve fibers, thereby inducing long-lasting analgesia. Additionally, Hausdorf et al. applied a moderate level of energy (1500 pulses with 0.9 mJ/mm^2^) to the hind limbs of rabbits, which led to a loss in the unmyelinated nerve within the femoral nerve of the treated limb [72]. Previous studies also indicate that intraprostatic capsaicin injection in rats triggers the stimulation of C-afferent fibers, leading to increased expressions of cyclooxygenase-2, NGF, and other inflammatory mediators within the prostate. LiESWT effectively reduced the induced pain behaviors in a manner that was contingent on both the treatment duration and dosage [70,73]. Our previous findings demonstrate that the therapeutic effectiveness of LiESWT could enhance voided volume and alleviate detrusor hyperactivity induced by OHD in a rat model.

We used OVX-treated rats to mimic the physiological conditions of OHD, or the postmenopausal state, to induce detrusor hyperactivity symptoms [74,75]. The influence of postmenopausal hypoestrogen on bladder dysfunction in OVX animals can be mediated by multiple factors, including neural control, vascular supply, detrusor muscle cell size and number, and connective tissue density and distribution. In a rat model of OHD-induced DHIC, the issue of LiESWT in improving the symptoms of detrusor hyperactivity via TRPV channels involved in nociception and mechanosensation during bladder filling is still controversial. We hypothesized that LiESWT has a therapeutic effect on bladder angiogenesis, neurogenesis, and detrusor muscle contraction via TRPV channels that involve calcium signaling, thereby improving detrusor hyperactivity. We also assessed whether LiESWT affects the expressions of TRPV1 and TRPV4 by studying the calcium signaling pathway. The experimental design is shown in Table 1. We investigated whether LiESWT modulates the generation of Ca^2+^ oscillation and the activation of ER-related Ca^2+^ channels/receptors in the ER membrane, as well as anoctamin-1 (Ano1) channels in the plasma membrane, to promote detrusor muscle contraction. Furthermore, we quantified the expressions of signaling-related proteins after LiESWT treatment, including Gq/11, Gq/12, Gq/13, RhoA, and RhoK, which are involved in the activation of ER-related Ca^2+^ channels/receptors, to modulate the generation of Ca^2+^ oscillation.

## 2. Results

### 2.1. Serum Parameters Were Reduced after OVX

OVX-treated rats were used to mimic menopausal status with OHD. The serum estradiol concentrations at one month following the OVX surgery are presented in Table 2. In comparison with the sham group (33.5 ± 3.4 pg/mL), the serum estradiol levels significantly decreased by 16.4 ± 1.3 pg/mL in the OVX group; 15.6 ± 1.0 pg/mL in the OHD status for 12 months, followed by once weekly LiESWT for 4 weeks (OVX + SW4) group; and 15.5 ± 1.4 pg/mL in the OHD status for 12 months, followed by twice weekly LiESWT for 4 weeks (OVX + SW8) group. The results show that estradiol deficiency was induced by bilateral OVX surgery.

Serum calcium and phosphate concentrations are shown in Table 2. Contrasted with the sham group’s serum calcium level of 10.5 ± 0.3 mg/dL, the OVX group exhibited a notable decrease of 9.4 ± 0.4 mg/dL. Similarly, the serum calcium levels slightly dropped to 10.2 ± 0.5 mg/dL in the OVX + SW4 group and to 10.5 ± 0.3 mg/dL in the OVX + SW8 group. Moreover, the serum phosphate levels meaningfully decreased by 3.7 ± 0.7 mg/dL in the OVX group, 4.7 ± 1.0 mg/dL in the OVX + SW4 group, and 4.3 ± 0.6 mg/dL in the OVX + SW8 group in contrast to the sham group (5.3 ± 0.8 mg/dL). On the basis of these results, the levels of the serum calcium and phosphate concentrations were reduced in the OVX group, whereas the levels in the OVX + SW4 group and the OVX + SW8 group were similar to the sham group. However, there was no significant difference in the ratio of serum calcium level/phosphate level among the different groups.

The association between the elevated phosphate and calcium levels is essential for normal neuromuscular function. The diagnostic value of the serum calcium level to phosphate level (Ca/P) ratio in the diagnosis of detrusor hyperactivity was induced by OHD. However, the serum Ca/P ratio was not a valuable tool for the diagnosis of detrusor hyperactivity induced by OHD.

### 2.2. Physical Characteristics

Their physical characteristics after 12 months of bilateral OVX are detailed in Table 2, encompassing parameters such as water intake, urine output, body weight, bladder weight, and the ratio of bladder weight to body weight. No significant differences were observed in terms of water intake, urine output, and bladder weight among the four groups. However, in the OVX group, there was a meaningful increase in body weight, whereas the ratio of bladder weight to body weight was significantly reduced compared with the sham group. In addition, LiESWT in the OVX + SW4 group and the OVX + SW8 group resulted in a slight increase in the ratio of bladder weight to body weight, and it showed limited restoration of body weight at the control level. These results indicate that OHD had a profoundly negative effect on body weight and the ratio of bladder weight to body weight, which resulted in the pathological alteration in the bladder.

### 2.3. LiESWT Treatment Ameliorated Bladder Hyperactivity

**Figure 1:**
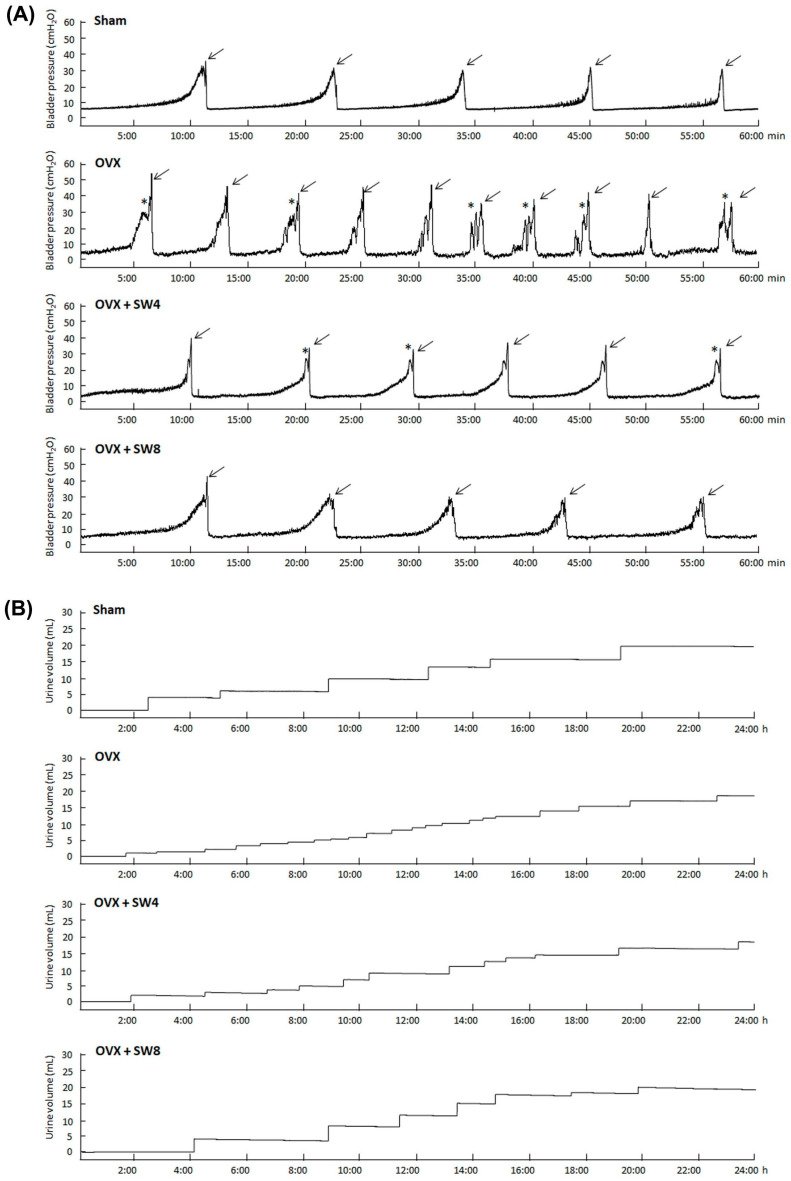
LiESWT improved voiding behavior and ameliorated bladder detrusor hyperactivity in a rat model of OHD-induced DHIC: (**A**) urodynamic analysis of cystometric parameters, including micturition pressure, voiding frequency, volume, contraction (arrows), and nonvoiding contraction (asterisks) in the different groups; (**B**) tracing analysis of 24-h voiding behavior by metabolic cage in the different groups. The OVX group exhibited increased bladder maturation pressure, voiding contraction, nonvoiding contraction, and micturition frequency, while the LiESWT groups had improved bladder voiding patterns and volumes. LiESWT, low-intensity extracorporeal shock wave therapy; DHIC, detrusor hyperactivity with impaired contractility; OHD, ovarian hormone deficiency; OVX, bilateral ovariectomy; OVX + SW4, OHD status for 12 months, followed by once weekly LiESWT for 4 weeks; OVX + SW8, OHD status for 12 months, followed by twice weekly LiESWT for 4 weeks.

Bladder function was assessed using urodynamic parameters and voiding behavior, which encompassed peak micturition pressure, micturition frequency, micturition interval, voided volume, and nonvoided contraction (asterisks). The results are presented in Table 2 and Figure 1. The cystometrogram (CMG) data from the sham group illustrate a consistent and steady micturation pattern, whereas the OVX group exhibited bladder hyperactivity, characterized by an elevated micturition frequency (arrows), nonvoiding contractions (asterisks), and decreased micturition volume. On the contrary, both the OVX + SW4 group and the OVX + SW8 group exhibited notable reductions in frequency and heightened micturition volumes compared with the OVX group (Table 2 and Figure 1A). However, there were no meaningful differences among the different groups in the peak micturition pressure.

From an analysis of the micturition behavior, the OVX group had a lower voided volume and greater micturition frequency than the sham group (Figure 1B). However, both the OVX + SW4 group and the OVX + SW8 group exhibited significant reductions in micturition frequency while displaying increased voided volumes compared with those in the OVX group. Therefore, the LiESWT contributed to the enhancement of voiding behavior and the amelioration of bladder overactivity. Taken together, the results suggest that the OVX-treated rats exhibited significant bladder hyperactivity, abnormal detrusor activity with an increase in the micturition frequency, and deteriorated micturition volumes, whereas the administration of LiESWT led to noteworthy enhancements in micturition volumes and alleviated the symptoms of detrusor hyperactivity induced by OHD.

### 2.4. LiESWT Treatment Ameliorated Bladder Detrusor Contractile Response

**Figure 2:**
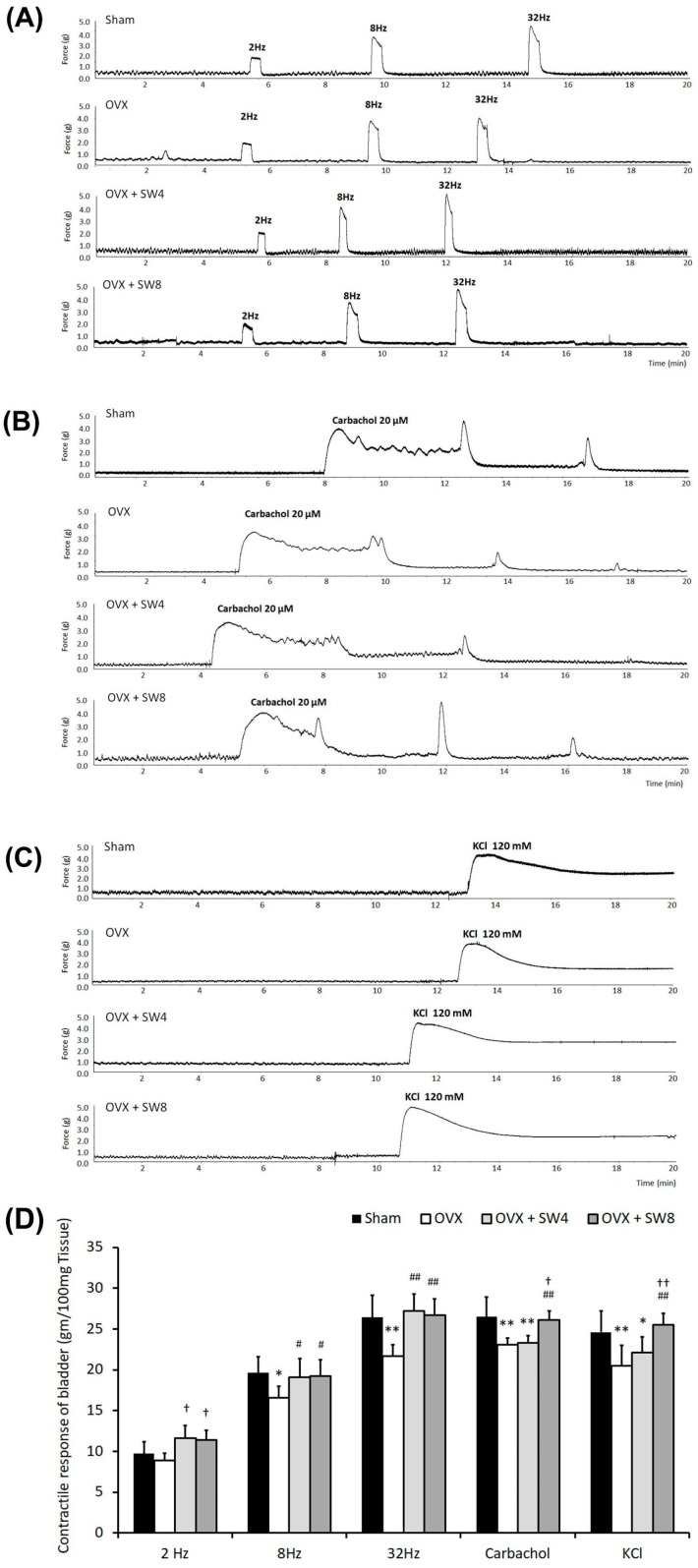
Contractile responses of bladder strips to EFS, carbachol, and KCl. After 12 months of OVX, bladder strips had lower EFS-induced contractile responses at 2, 8, and 32 Hz than the sham group. However, the OVX + SW8 groups had higher EFS-induced contractile responses at 8 and 32 Hz compared with the (**A**,**D**) OVX group. Treatment with (**B**,**D**) carbachol and (**C**,**D**) KCl in the OVX group induced lower contractile responses than the sham group, while the contractile responses were enhanced in the OVX + SW4 group and OVX + SW8 group. The LiESWT ameliorated bladder detrusor contractile responses by using muscle strips for synaptic transmission, receptor response, and smooth muscle contraction. EFS, electrical-field stimulation; OVX, bilateral ovariectomy; DHIC, detrusor hyperactivity with impaired contractility; LiESWT, low-intensity extracorporeal shock wave therapy; OVX + SW4, OHD status for 12 months, followed by once weekly LiESWT for 4 weeks; OVX + SW8, OHD status for 12 months, followed by twice weekly LiESWT for 4 weeks. Data are expressed as the means ± SD for n = 6. * *p* < 0.05 and ** *p* < 0.01 versus the sham group; ^#^ *p* < 0.05 and ^##^ *p* < 0.01 versus the OVX group; ^†^ *p* < 0.05 and ^††^ *p* < 0.01 versus the OVX + SW4 group.

The contractile response of the bladder detrusor was evaluated in terms of synaptic transmission, receptor activity, and smooth muscle contraction. The results of the electrical-field, carbachol, and KCl stimulations for contractile responses on the bladder strips are shown in Figure 2. The bladder strips in the OVX group had lower contractile responses induced by electrical-field stimulation (EFS) at 2, 8, and 32 Hz compared with the sham group, while the OVX + SW4 group and the OVX + SW8 group had higher contractile responses compared with the OVX group (Figure 2A,D). Similar results were obtained for the muscle strip stimulation induced by carbachol (Figure 2B,D) and KCl (Figure 2C,D). The LiESWT treatment ameliorated the bladder detrusor contractile response using muscle strips for synaptic transmission, receptor response, and smooth muscle contraction. These results indicate that the OVX rats had worse bladder contractile responses, which caused bladder contractile deficiency, while the LiESWT ameliorated the bladder contractile function.

### 2.5. LiESWT Improved OVX-Induced Pathological Alteration, Altered Bladder Angiogenic Remodeling, and Interstitial CELL (IC) Generation

**Figure 3:**
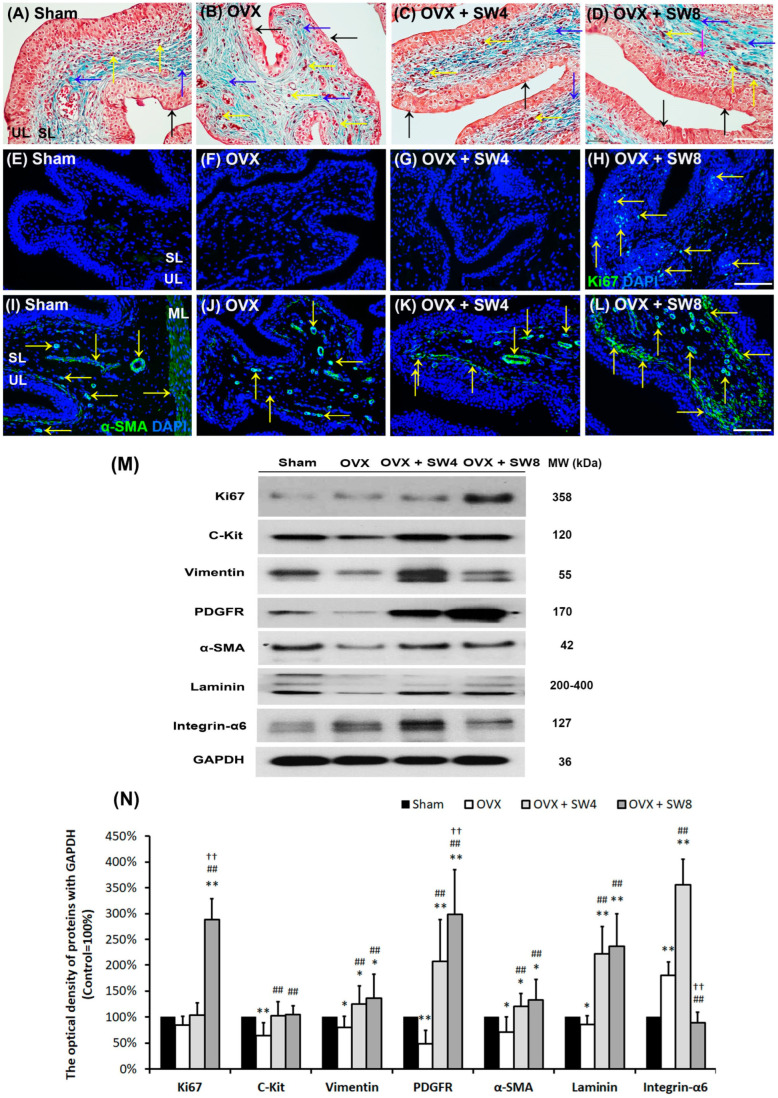
Therapeutic effects of the LiESWT improved OHD-induced pathological alteration, angiogenesis remodeling, and interstitial cell generation to modulate muscle contraction. Masson’s trichrome staining, immunostaining, and Western blots were used to examine the pathological alteration, angiogenesis, and IC generation. Nuclear DNA was labeled with DAPI (blue). (**A**–**D**) Bladder pathological features of the (**A**) sham group, (**B**) OVX group, (**C**) OVX + SW4 group, and (**D**) OVX + SW8 group. Masson’s trichrome stain showed red-stained smooth muscle and green-stained collagen. In the (**A**) sham group, there were three to five layers of UL (black arrows), sparse collagen (blue arrows), and ICs (yellow arrows) distributed in the SL (lamina propria). In the (**B**) OVX group, the morphology was characterized with a thinner layer of UL (black arrows), decreased IC generation (yellow arrows), and increased interstitial fibrosis (blue arrows). In contrast, the pathological features of the (**C**) OVX + SW4 group and the (**D**) OVX + SW8 group showed improved OHD-induced bladder damages by increasing thicker UL (black arrows), ICs (yellow arrows), and reducing interstitial fibrosis (blue arrows) compared with the OVX group. The myofibroblastic phenotype and localization were evaluated by immunostaining the expression of Ki67, α-SMA, laminin, and vimentin. (**E**–**H**) The distribution of Ki67 for cell proliferation was shown by immunostaining, and the staining of the proliferation marker Ki67 showed less distribution in the bladder tissues of the (**E**) sham group, (**F**) OVX group, and (**G**) OVX + SW4 group. On the contrary, the Ki67 immunostaining was obviously expressed in the urothelial basal layer and the sphere of the SL in the (**H**) OVX + SW8 group. (**I**–**L**) The distribution of α-SMA for angiogenesis was shown by immunostaining. In the (**I**) sham group, α-SMA was abundantly expressed on the microvasculature at the SL and ML, while the staining decreased in the SL and ML of the (**J**) OVX group. The immunostaining of the (**K**) OVX + SW4 group and (**L**) OVX + SW8 group showed an enhancement of the expression. Particularly, there were many gathered α-SMA-positive myofibroblasts and microvessels (yellow arrows) beneath the urothelial basal layer and lamina propia in the OVX + SW8 group. (**M**,**N**) The protein levels of cell proliferation (Ki67), IC markers (C-Kit, vimentin, and PDGFR), and angiogenesis (α-SMA, laminin, and integrin-α6) were evaluated by Western blot analysis. The levels of α-SMA, laminin, and IC markers significantly decreased in the OVX group compared with the sham group, except integrin-α6. However, the levels noticeably increased in the OVX + SW4 group and OVX + SW8 group compared with the OVX group. OVX, bilateral ovariectomy; OHD, ovarian hormone deficiency; LiESWT, low-intensity extracorporeal shock wave therapy; OVX + SW4, OHD status for 12 months, followed by once weekly LiESWT for 4 weeks; OVX + SW8, OHD status for 12 months, followed by twice weekly LiESWT for 4 weeks; DHIC, detrusor hyperactivity with impaired contractility; DAPI, 4′,6-diamidino-2-phenylindole; α-SMA, α-smooth muscle actin; GAPDH, glyceraldehyde-3-phosphate dehydrogenase; PDGFR, platelet-derived growth factor receptor; IC, interstitial cell; SL, suburothelial layer; ML, muscular layer; UL, urothelial layer. Data are expressed as the means ± SD for n = 8. * *p* < 0.05 and ** *p* < 0.01 versus the sham group; ^##^ *p* < 0.01 versus the OVX group; ^††^ *p* < 0.01 versus the OVX + SW4 group.

To elucidate whether LiESWT improved angiogenesis and IC generation in the bladder in a rat model of OHD-induced detrusor hyperactivity, the pathological changes, cell-proliferating proteins (Ki67), angiogenesis-related markers (α-SMA, Laminin and integrin-α6) and IC markers (C-Kit, vimentin and PDGFR) were quantified by Masson’s trichrome staining (Figure 3A–D), immunostaining (Figure 3E–H), and Western blots (Figure 3I,J). Masson’s trichrome stain was employed to examine pathological alterations in the bladder following treatment (Figure 3A–D). In the sham group (Figure 3A), the urothelial layer (UL; black arrows) was composed of three to five layers, ICs (yellow arrows), and sparse collagen fibers (black arrowhead) were found within the suburothelial layer (SL). On the contrary, in the OVX group, bladders displayed a thinner and compromised urothelial mucosa in the UL (black arrows), ICs (yellow arrows) and interstitial fibrosis (blue arrows). Similarly, there were ICs (yellow arrows) and interstitial fibrosis (blue arrows) in the SL of the OVX + SW4 group and the OVX + SW8 group (Figure 3C,D). However, the morphological assessment of the OVX + SW8 group (Figure 3D) revealed a notable improvement in bladder damage caused by OVX because of the presence of a thicker UL and the regulation of IC proliferation (purple arrow), along with the mitigation of collagen accumulation (blue arrows).

The myofibroblastic phenotype was assessed through both immunostaining and a Western blot analysis of the Ki67, α-SMA, laminin, and vimentin expressions. The distribution of the proliferation marker Ki67 was less prominent in the bladder tissues of the sham group, OVX group, and OVX + SW4 group. Conversely, the Ki67 immunostaining was notably evident in the urothelial basal layer and the sphere of the SL in the OVX + SW8 group (Figure 3E–H). In the sham group (Figure 3I), the immunostaining of α-SMA (yellow arrows) exhibited a broad distribution within the myofibroblasts and smooth muscle of microvessels beneath the urothelial basal layer in the SL (lamina propria) and ML, respectively. In the OVX group (Figure 3J), the immunostaining of α-SMA was diminished in comparison with the sham group. However, the expressions were heightened in myofibroblasts within microvessels and vessels in the SL and ML of both the OVX + SW4 group and the OVX + SW8 group compared with the OVX group (Figure 3K,L). Particularly, the OVX + SW8 group exhibited a notable presence of clustered α-SMA-positive myofibroblasts and microvessels (yellow arrows) beneath the urothelial basal layer within the SL and ML (Figure 3H).

Western blotting analysis was utilized to conduct a more comprehensive examination of the protein proliferation levels (Ki67) (Figure 3M,N). The protein level of Ki67 was meaningfully enhanced in the OVX + SW8 group in comparison with both the OVX group and the OVX + SW4 group. Moreover, the levels of C-Kit, vimentin, PDGFR, α-SMA, and laminin, as observed in the OVX group, significantly declined. Conversely, the expression of integrin-α6 demonstrated a meaningful enhancement in comparison with the sham group. Moreover, the levels of C-Kit, vimentin, PDGFR, α-SMA, and laminin were obviously elevated in both the OVX + SW4 group and the OVX + SW8 group when compared with the OVX group, except the expression of integrin-α6 (Figure 3M,N). According to the above data, the LiESWT improved the urothelial proliferation and stimulated Ki67^+^-associated fibroblasts in the SL to modulate fibroblast recruitment and improve mucosal regeneration. The LiESWT also increased IC generation and altered bladder angiogenic remodeling for bladder repair in the pathogenesis of OHD-induced detrusor hyperactivity.

### 2.6. LiESWT Promoted Bladder Neurogenesis in OVX-Induced Detrusor Hyperactivity

**Figure 4:**
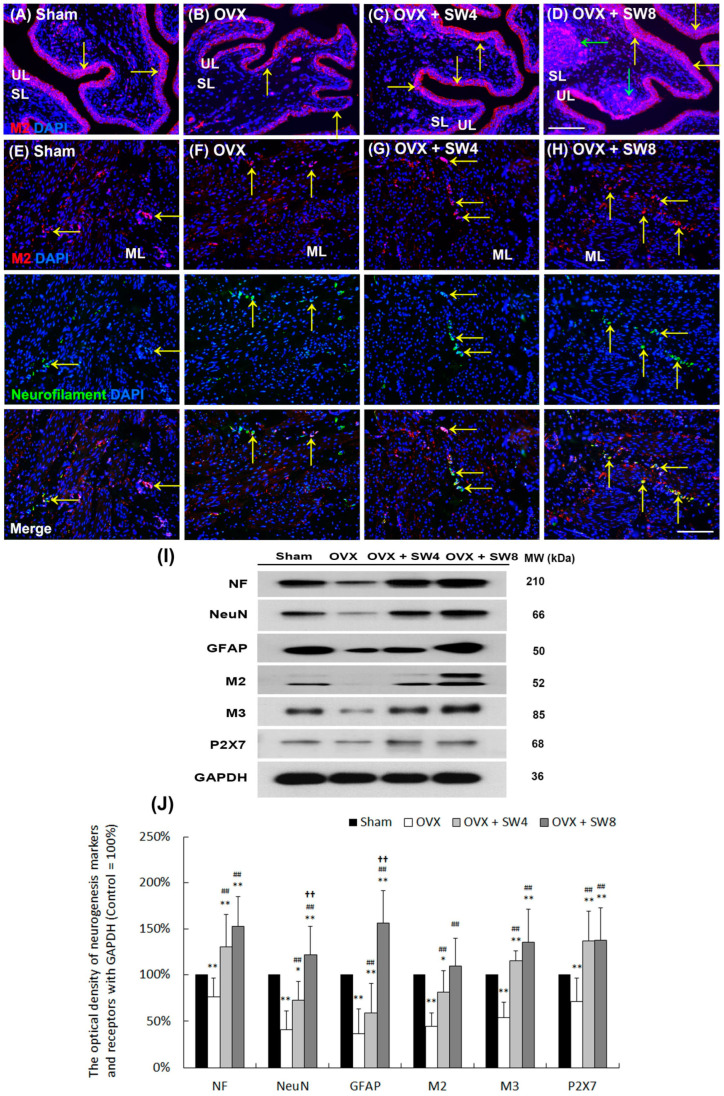
LiESWT increased neuronal regeneration, synaptic transmission, and receptor response. The expressions of neuronal endogenous markers (NF, NeuN, and GFAP) and muscarinic receptor (M2 and M3), and purinergic receptor (P2X7) markers were assessed by immunostaining (**A**–**H**) and Western blots (**I**,**J**). Nuclear DNA was labeled with DAPI (blue). (**A**–**D**) The M2 immunostaining was markedly expressed in the UL and SL of the (**A**) sham group. On the contrary, there was less M2 staining expression in the thinner and defective urothelial mucosa in the UL (yellow arrows) of the (**B**) OVX group, but the immunostainings in the (**C**) OVX + SW4 group and the (**D**) OVX + SW8 group were enhanced. (**E**–**H**) Double-labeled analysis of M2 (red, upper panels) and NF (green, lower panels) was distributed in the ML of the (**E**) sham group. However, the double staining of the (**G**) OVX + SW4 group and the (**H**) OVX + SW8 group were widely expressed compared with the (**F**) OVX group. (**I**,**J**) Quantifications of the percentage of neurogenesis-related markers, muscarinic receptors, and purinergic receptors were evaluated by Western blotting. The expressions obviously decreased in the OVX group compared with the sham group. However, the expressions significantly increased in the OVX + SW4 group and OVX + SW8 group compared with the OVX group. Therefore, the LiESWT promoted bladder synaptic transmission, receptor response, and neurogenesis to ameliorate the bladder detrusor contractile response. Nuclear DNA was labeled with DAPI (blue). LiESWT, low-intensity extracorporeal shock wave therapy; OVX + SW4, OHD status for 12 months, followed by once weekly LiESWT for 4 weeks; OVX + SW8, OHD status for 12 months, followed by twice weekly LiESWT for 4 weeks; DAPI, 4′,6-diamidino-2-phenylindole; NF, neurofilament; NeuN, neuronal nuclear antigen and neuron; GFAP, glial fibrillary acidic protein; GAPDH, glyceraldehyde-3-phosphate dehydrogenase; UL, urothelial layer; SL, suburothelial layer. Data are expressed as the means ± SD for n = 8. * *p* < 0.05 and ** *p* < 0.01 versus the sham group; ^##^ *p* < 0.01 versus the OVX group; ^††^ *p* < 0.01 versus the OVX + SW4 group.

To investigate the effect of LiESWT on bladder neurogenesis, including neuronal regeneration, synaptic transmission, and receptor response, the expressions of neuronal endogenous markers (neurofilament (NF), neuronal nuclei (NeuN), and glial fibrillary acidic protein (GFAP)], muscarinic receptor (M2 and M3), and purinergic receptor (P2X7)) were assessed by immunostaining and Western blots (Figure 4). In the sham group (Figure 4A), the M2 staining (yellow arrows) was mainly expressed in the UL. Compared with the sham group, the OVX group exhibited reduced M2 staining (yellow arrows) primarily localized to the thinner and disrupted urothelium (Figure 4B). However, in contrast to the OVX group, the OVX + SW4 group and the OVX + SW8 group displayed a more extensive distribution of M2 staining (yellow arrows) within the UL (Figure 4C,D). Particularly, the labeling of the OVX + SW8 group exhibited prominent expressions in the urothelial basal layer and the sphere of the SL (lamina propria) (green arrows) compared with the OVX group (Figure 4D). Moreover, the costaining of M2 and NF (yellow arrows) was distributed within the ML in the sham group (Figure 4E). In the OVX group (Figure 4F), the costaining (yellow arrows) was significantly suppressed compared with the sham group. However, the costaining (yellow arrows) was enhanced in the OVX + SW4 group and OVX + SW8 group (Figure 4G,H).

For the Western blot analysis, the markers of mature neuron, glial cell, muscarinic, and purinergic receptors were found to be significantly suppressed in the OVX group compared with the sham group, whereas the expressions meaningfully increased in the OVX + SW4 group and the OVX + SW8 group compared with the OVX group (Figure 4I,J). The results of the morphological evaluation and Western blot for OHD after 12 months of OVX revealed neuronal degeneration, while LiESWT could enhance neurogenesis and receptor responses to improve bladder overactivity.

### 2.7. LiESWT Altered TRPV Channel Expression in Bladder

**Figure 5:**
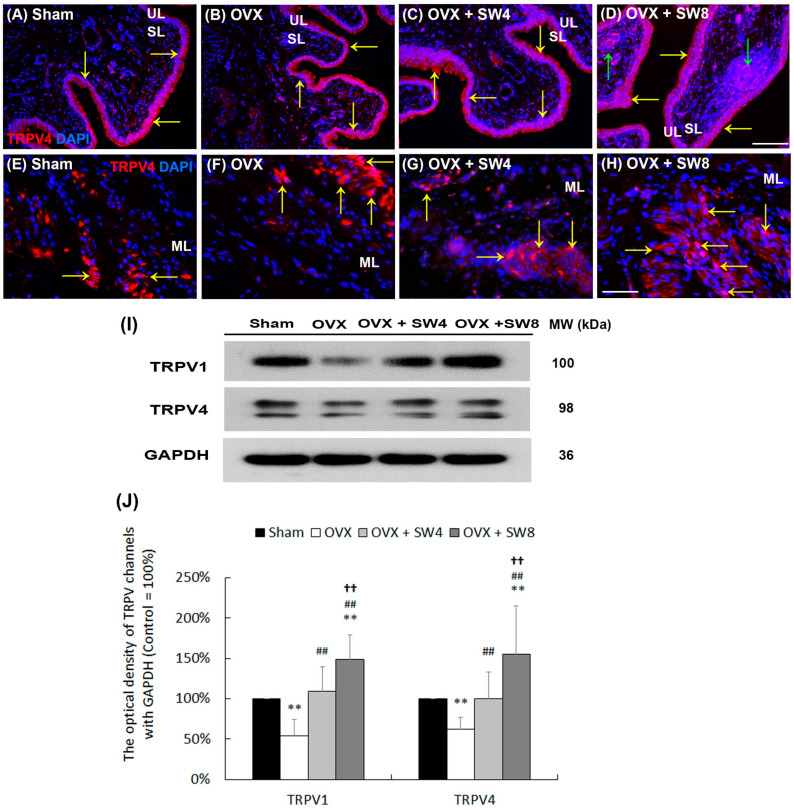
The effect of the LiESWT was an enhanced TRPV expression. The expressions of the TRPV channels were shown using (**A**–**H**) immunostaining and (**I**,**J**) Western blots. (**A**–**H**) The distributions of the TRPV4 channel were observed in the (**A**–**D**) UL and (**E**–**H**) ML. Nuclear DNA was labeled with DAPI (blue). Immunostaining of TRPV4 (red) in the sham group showed abundant staining in the (**A**) UL (yellow arrows) and some staining in the € ML(yellow arrows). In contrast, the expressions of the TRPV4 staining in the OVX group were lower in the thinner (**B**) UL and (**F**) ML. However, the immunostaining of the (**C**,**G**) OVX + SW4 group and the (**D**,**H**) OVX + SW8 group showed enhanced staining in the thick UL, SL, and ML. (**I**,**J**) Western blot was performed to evaluate the protein levels of TRPV1 and TRPV4. Both proteins significantly decreased in the OVX group compared with the sham group. However, the expressions noticeably increased in the OVX + SW4 group and the OVX + SW8 group compared with the OVX group. Besides, the labeling of the OVX + SW8 group exhibited prominent expressions in the urothelial basal layer and the sphere of the SL (green arrows) compared with the OVX group. LiESWT, low-intensity extracorporeal shock wave therapy; OVX, bilateral ovariectomy; OVX + SW4, OHD status for 12 months, followed by once weekly LiESWT for 4 weeks; OVX + SW8, OHD status for 12 months, followed by twice weekly LiESWT for 4 weeks; DAPI, 4′,6-diamidino-2-phenylindole; TRPV, transient receptor potential vanilloid; GAPDH, glyceraldehyde-3-phosphate dehydrogenase; UL, urothelial layer; SL, suburothelial layer; ML, muscular layer. Data are expressed as the means ± SD for n = 8. ** *p* < 0.01 versus the sham group; ^##^ *p* < 0.01 versus the OVX group; ^††^ *p* < 0.01 versus the OVX + SW4 group.

To further explore whether the effect of LiESWT improves the symptoms of detrusor hyperactivity via TRPV channels involving nociception and mechanosensory transduction to modulate the calcium concentration in OHD-induced detrusor hyperactivity, the expressions of TRPV1 and TRPV4 were examined by immunofluorescence and Western blot (Figure 5). In the bladder of the sham group, the immunostaining of the TRPV4 channels was not only abundantly expressed in the UL (Figure 5A) but may also have been localized in the SL and ML (Figure 5E). Within the OVX group (Figure 5B,F), the TRPV4 staining (yellow arrows) was confined to the urothelium, SL, and ML, which appeared thinner and disrupted when compared with the sham group. However, in the OVX + SW4 group and the OVX + SW8 group, the expressions were significantly enhanced in the UL (Figure 5B), SL, and ML (Figure 5F). Besides, the labeling of the OVX + SW8 group exhibited prominent expressions in the urothelial basal layer and the sphere of the SL (green arrows) compared with the OVX group (Figure 5D).

Western blot was further used to evaluate the protein levels of the TRPV1 and TRPV4. In the OVX group, the levels were reduced compared with the sham group (Figure 5I,J). After the LiESWT, there were significant increases in the levels of both TRPV1 and TRPV4 in the OVX + SW4 group and the OVX + SW8 group compared with the OVX group. The expressions in the OVX + SW8 group were as profound as those in the sham group. On the basis of the data from the morphological evaluation and Western blot, the OVX group had meaningfully reduced levels of TRPV1 and TRPV4. In contrast, the LiESWT altered the TRPV channel expression to modulate the calcium concentration and increase the micturition volume, as well as ameliorate urinary frequency with bladder hyperactivity.

### 2.8. LiESWT Altered the ER-Related Calcium Receptors

**Figure 6:**
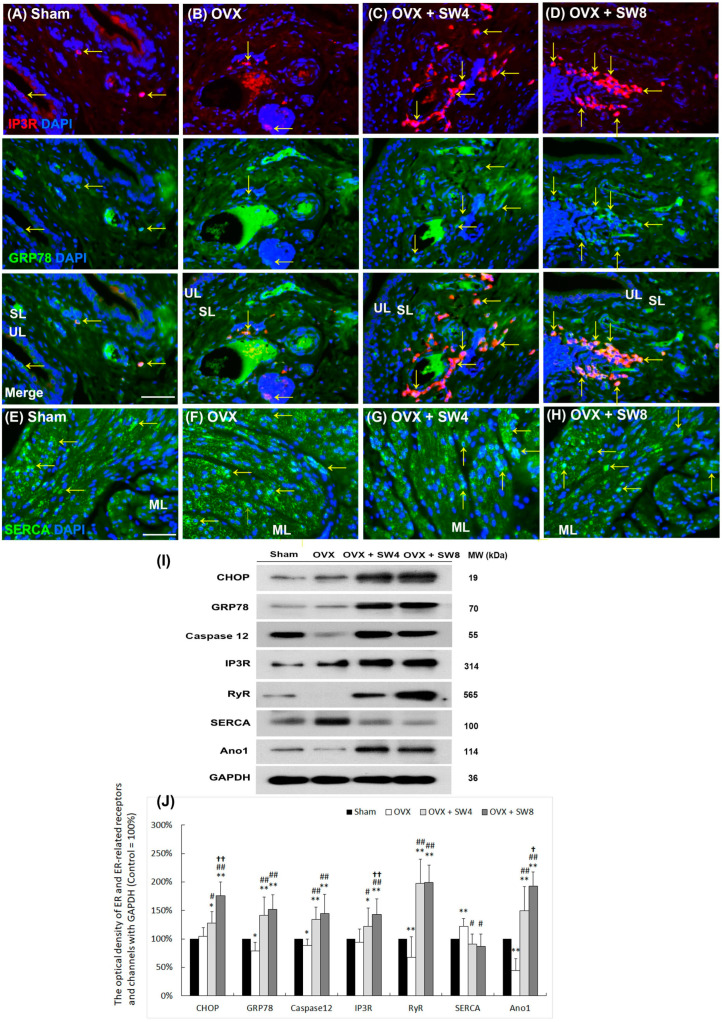
LiESWT activated ER-related Ca^2+^ channels/receptors to modulate the generation of the Ca^2+^ level for detrusor muscle contraction. The expressions of the ER stress protein (CHOP, GRP78, and caspase 12), ER-related Ca^2+^ channels and receptors (RyR, IP3R, and SERCA), and Ano1 were assessed by immunostaining (**A**–**H**) and Western blots (**I**,**J**). Nuclear DNA was labeled with DAPI (blue). (**A**–**D**) From the double-labeled analysis, the IP3R (red, upper panels) and GRP78 (green, lower panels) were slightly distributed in the UL and SL of the (**A**) sham group. However, the double staining (yellow arrows) of the (**C**) OVX + SW4 group and the (**D**) OVX + SW8 group were markedly expressed in the SL compared with the (**B**) OVX group. (**E**–**H**) The SERCA immunostaining (green, yellow arrows, (**E**–**H**)) was distributed in the ML of the (**E**) sham group. However, the staining in the ML of the (**F**) OVX group was performed in comparison with the sham group. The SERCA expressions declined in the ML of the (**G**) OVX + SW4 group and the (H) OVX + SW8 group compared with the OVX group. (**I**,**J**) The quantities of ER stress protein and ER-related Ca^2+^ channels/receptors were evaluated by Western blotting. LiESWT enhanced the expression of ER stress proteins and stimulated ER-related Ca^2+^ channels/receptors to modulate the calcium level. Moreover, the expression of SERCA increased in the OVX group compared with the sham group, while it declined in the OVX + SW4 group and the OVX + SW8 group. LiESWT, low-intensity extracorporeal shock wave therapy; OVX, bilateral ovariectomy; OVX + SW4, OHD status for 12 months, followed by once weekly LiESWT for 4 weeks; OVX + SW8, OHD status for 12 months, followed by twice weekly LiESWT for 4 weeks; DAPI, 4′,6-diamidino-2-phenylindole; ER, endoplasmic reticulum; CHOP, C/EBP homologous protein; GRP 78, glucose-regulated protein 78; RyRs, ryanodine receptors; IP3Rs, inositol triphosphate receptors; SERCA, sarco/endoplasmic reticulum Ca^2+^-ATPase; GAPDH, glyceraldehyde-3-phosphate dehydrogenase; UL, urothelial layer; SL, suburothelial layer. Data are expressed as the means ± SD for n = 8. * *p* < 0.05 and ** *p* < 0.01 versus the sham group; ^#^ *p* < 0.05 and ^##^ *p* < 0.01 versus the OVX group; ^†^ *p* < 0.05 and ^††^ *p* < 0.01 versus the OVX + SW4 group.

To further explore the therapeutic efficacy of LiESWT, the ER stress protein [C/EBP homologous protein (CHOP), glucose-regulated protein 78 (GRP 78), and caspase 12] and ER-related Ca^2+^ channels and receptors [ryanodine receptors (RyRs), inositol triphosphate receptors (IP3Rs), sarco/endoplasmic reticulum Ca^2+^-ATPase (SERCA), and Ano1] for detrusor muscle contractility, their expressions were evaluated by immunostaining and Western blots (Figure 6). The costaining (yellow arrows) of GRP78 and IP3R displayed a comparatively reduced distribution within the UL, SL, and ML both in the sham group and the OVX group. The costaining was obviously expressed within the UL, SL, and ML of both the OVX + SW4 group and the OVX + SW8 group compared with the OVX group (Figure 6A–D). Moreover, the SERCA staining was distributed in the ML of the sham group. However, the staining in the ML of the OVX group was obviously shown in comparison with the sham group. The SERCA expressions declined in the ML of the OVX + SW4 group and the OVX + SW8 group compared with the OVX group (Figure 6E–H).

Western blot analysis was performed to further investigate the levels of ER stress proteins and ER-related Ca^2+^ channels and receptors (Figure 6I,J). The protein levels of GRP78, caspase 12, RyR, and Ano1 significantly decreased in the OVX group in comparison with the sham group, except CHOP, SERCA, and IP3R. However, these protein levels were obviously enhanced in the OVX + SW4 group and the OVX + SW8 group compared with the OVX group, except SERCA. On the contrary, the expression of SERCA increased in the OVX group compared with the sham group, which obviously declined in the OVX + SW4 group and the OVX + SW8 group. The costaining protein expression in the OVX + SW4 group was as profound as in the OVX + SW8 group. These observations suggest that LiESWT could enhance the expression of ER stress proteins and stimulate ER-related Ca^2+^ channels and receptors to modulate calcium levels.

### 2.9. Cellular Signaling Pathway Involved in Regulating Intracellular Ca^2+^ Oscillation in A Rat Model of OHD-induced DHIC

**Figure 7:**
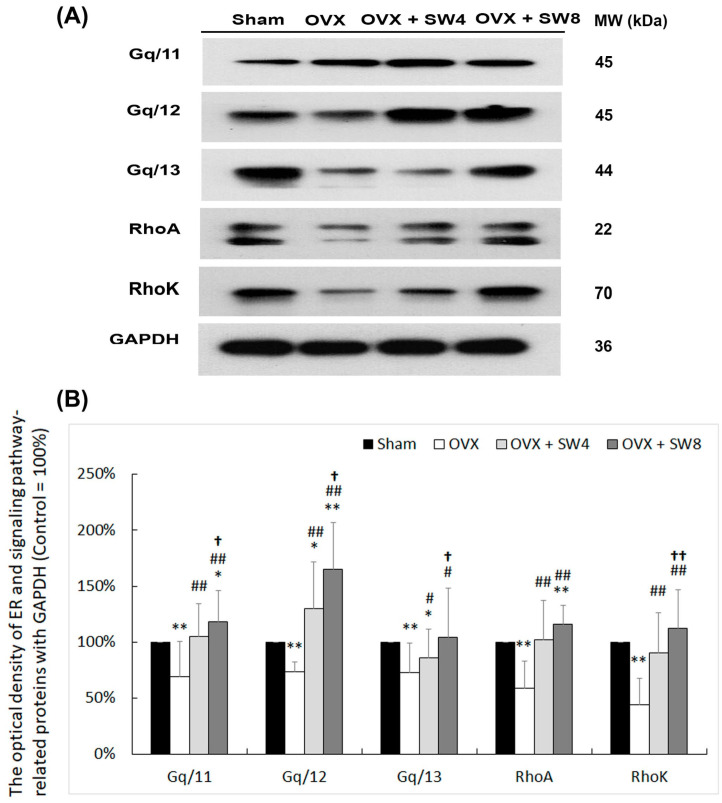
The cellular signaling pathway involved in regulating intracellular Ca^2+^ oscillation in a rat model of OHD-induced DHIC. The expressions of signaling-pathway-related proteins, including Gq/11, Gq/12, Gq/13, RhoA, and RhoK, were quantified by Western blots (**A**). In the OVX group, the protein expression levels were reduced compared with the sham group. The LiESWT treatment significantly promoted the protein levels in the OVX + SW4 group and the OVX + SW8 group in comparison with the OVX group. DHIC, detrusor hyperactivity with impaired contractility. Data are expressed as the means ± SD for n = 8. * *p* < 0.05 and ** *p* < 0.01 versus the sham group; ^#^ *p* < 0.05 and ^##^ *p* < 0.01 versus the OVX group; ^†^ *p* < 0.05 and ^††^ *p* < 0.01 versus the OVX + SW4 group (**B**).

To elucidate whether the effects of the LiESWT improved the symptoms of detrusor hyperactivity via the cellular signaling pathway involved in regulating intracellular Ca^2+^ oscillation, the expressions of signaling-pathway-related proteins, including Gq/11, Gq/12, Gq/13, RhoA, and RhoK, were quantified by Western blot (Figure 7). In the OVX group, the expression levels were diminished in comparison with the sham group. However, treatment with LiESWT substantially elevated the expression levels in both the OVX + SW4 group and the OVX + SW8 group when compared with the expression levels observed in the OVX group. Particularly, the protein levels of the OVX + SW8 group were significantly expressed compared with the OVX + SW4 group. On the basis of the above findings, we suggest that LiESWT modulates the intracellular calcium level through the cellular signaling pathway to improve OHD-induced detrusor hyperactivity.

### 2.10. A Proposed Diagram for the Effects of the LiESWT That Ameliorated Detrusor Hyperactivity with Impaired Contractility via Transient Potential Vanilloid Channels in A Rat Model of Ovarian Hormone Deficiency

**Figure 8:**
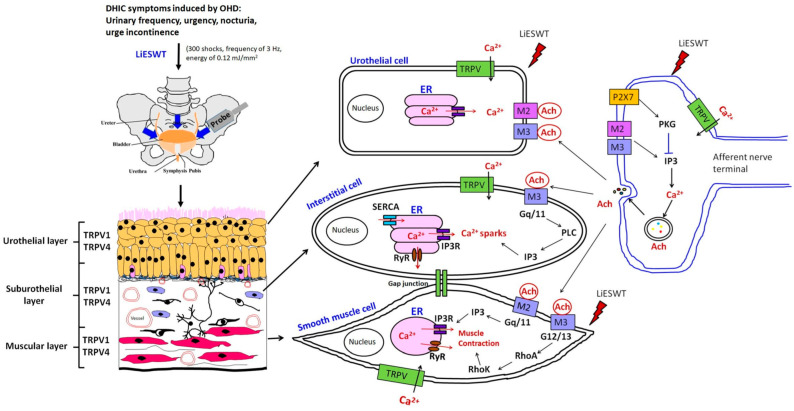
A brief diagram of the proposed therapeutic effect of the LiESWT in improving detrusor hyperactivity via TRPV channels and the cellular signaling pathway involved in regulating intracellular Ca^2+^ oscillation in a rat model of OHD-induced DHIC. The OVX-treated rat model was used to mimic the physiological conditions of OHD or the postmenopausal state to induce detrusor hyperactivity symptoms. LiESWT (0.12 mJ/mm^2^, 300 impulses, 3 Hz) was applied to the lower abdomen of the OHD-induced DHIC rats. Accordingly, the OVX rats had exacerbated pathological damage in the bladder and worse bladder contractile responses, while the LiESWT ameliorated OVX-induced detrusor hyperactivity and improved the bladder contractile function. Additionally, the neurogenesis effect of the LiESWT increased the levels of neurogenesis (NF, NeuN, and GFAP), muscarinic receptors (M2 and M3), purinergic receptor (P2X7), TRPV channels (TRPV1 and TRPV4), ER stress proteins (CHOP, GRP78, and caspase 12), ER-related Ca^2+^ channels/receptors (RyR, IP3R, and SERCA), and Ano1 that were involved in regulating intracellular Ca^2+^ oscillation. Meanwhile, the LiESWT treatment significantly enhanced the signaling-pathway-related proteins, including Gq/11, Gq/12, Gq/13, RhoA, and RhoK, in the bladder involved in the activation of ER-related Ca^2+^ channels/receptors. Therefore, the OVX rats had worse bladder contractile responses that caused bladder contractile deficiency in a rat model of OHD-induced DHIC, while the LiESWT reduced the detrusor hyperactivity and ameliorated the bladder contractile function. The above findings imply that the OHD status after 12 months of OVX resulted in neuronal degeneration and decreased activation of the TRPV1 and TRPV4 channels in a rat model of OHD-induced DHIC. Ach, acetylcholine; DHIC, detrusor hyperactivity with impaired contractility; ER, endoplasmic reticulum; IP3, inositol trisphosphate; IP3R, inositol trisphosphate receptor; LiESWT, low-intensity extracorporeal shock wave therapy; M2/M3, muscarinic receptors; OHD, ovary hormone deficiency; P2X7, purinergic receptor; PKG, protein kinase G; RhoA, ras homolog family member A; RhoK, Rho-associated protein kinase; RyR, ryanodine receptor; SERCA, sarcoplasmic/endoplasmic reticulum Ca-ATPase; TRPV, transient receptor potential vanilloid.

A brief diagram proposes the therapeutic effect of LiESWT-improved DHIC via TRPVs in a rat model of OHD (Figure 8). This proposed model establishes long-term OHD after 12 months of OVX and identifies the possible mechanism of the LiESWT treatment on OHD-induced DHIC. Accordingly, the OVX rats had exacerbated pathological damage in the bladder and worse bladder contractile responses, which caused bladder contractile deficiency, while the LiESWT improved the bladder contractile function. Additionally, the neurogenesis effect of the LiESWT increased the levels of neurogenesis (NF, NeuN, and GFAP), muscarinic receptors (M2 and M3), purinergic receptor (P2X7), TRPV channels (TRPV1 and TRPV4), ER stress proteins (CHOP, GRP78, and caspase 12), ER-related Ca^2+^ channels/receptors (RyR, IP3R, and SERCA), and Ano1. Meanwhile, LiESWT treatment significantly enhanced the signaling-pathway-related proteins, including Gq/11, Gq/12, Gq/13, RhoA, and RhoK, in the bladder involved in the activation of ER-related Ca^2+^ channels/receptors. Therefore, the OVX rats had worse bladder contractile responses which caused bladder contractile deficiency in a rat model of OHD-induced DHIC, while LiESWT reduced detrusor hyperactivity and ameliorated the bladder contractile function. 

## 3. Discussion

On the basis of the present findings, the OVX-treated rats exhibited significantly thinner and defective urothelial mucosa and abnormal detrusor activity, resulting in an elevated micturition frequency, nonvoiding contractions, and a decline in micturition volume. On the other hand, the LiESWT treatment led to a significant enhancement and a notable improvement in the symptoms associated with DHIC induced by OHD. The present study also revealed a decrease in the expressions of IC generation (C-Kit, vimentin, and PDGFR), angiogenesis (α-SMA and laminin), neurogenesis (NF, NeuN, and GFAP), muscarinic receptors (M2 and M3), purinergic receptor (P2X7), and TRPV channels (TRPV1 and TRPV4) in the OVX group as a result of DHIC. However, the effects of LiESWT significantly improved micturition volume, IC generation, angiogenesis remodeling, and neurogenesis, including neuronal regeneration, synaptic transmission, and receptor response. Moreover, the OHD conditions after 12 months of OVX-induced neuronal degeneration exhibited meaningful reductions in the expressions of TRPV1 and TRPV4 and decreased activations of ER-related Ca^2+^ channels/receptors (RyR, IP3R, SERCA, and Ano1). In contrast, the LiESWT enhanced neurogenesis and channel/receptor responses to ameliorate detrusor hyperactivity in a rat model of OHD-induced DHIC. The signaling-pathway-related proteins of the bladder involved in intracellular Ca^2+^ oscillation, including Gq/11, Gq/12, Gq/13, RhoA, and RhoK, were found to be reduced in the OVX group compared with the sham group. However, the LiESWT significantly promoted the expressions of these proteins. Therefore, the therapeutic effects of the LiESWT in improving long-term OHD-induced DHIC not only promoted angiogenesis, IC generation, and nerve regeneration, but also activated TRPV channels and ER-related Ca^2+^ channels/receptors through the cellular signaling pathway to regulate intracellular Ca^2+^ oscillation.

According to the previous literature, ovariectomized rats exhibit voiding dysfunction, including reduced voiding efficiency and detrusor hyperactivity and increased postvoiding residual urine [17]. Moreover, in female estrogen receptor β^−/−^ mice, the pathological morphology and contractile function of the bladder has shown urothelial ulceration, atrophy, and detrusor hyperactivity [18]. DHIC, conventionally known as DO with impaired contractility (DOIC), represents a form of voiding dysfunction commonly observed in the elderly population [76]. Clinically, the diagnosis of DHIC was established through urodynamic analysis, revealing distinctive features, such as involuntary contractions of the detrusor muscle, diminished compliance during bladder filling, a voiding pattern characterized by low pressure and reduced flow, as well as instances of urinary retention. In the present study, OVX rats exhibited thinner and compromised urothelial mucosa, along with the presence of interstitial fibrosis. Moreover, these OVX rats exhibited detrusor hyperactivity, characterized by increased voiding contractions, nonvoiding contractions, and increased frequency of micturition in contrast with the sham group. However, the LiESWT increased the micturition volume, decreased urinary frequency, and improved the bladder’s contractile function (Figure 1 and Figure 2). In addition, the bladder muscle strips had lower contractile responses in comparison with the sham group, while the OVX + SW4 group and the OVX + SW8 group had higher contractile responses compared with the OVX group (Figure 2), and there were no significant differences in peak micturition pressures across the various groups. 

Abarbanel and Marcus showed that the prevalence of DHIC among elderly patients reached up to 18% [4]. Although DHIC is associated with UUI, it does not significantly contribute to the UAB population. In a clinical context, DHIC is identified through urodynamic diagnosis, marked by involuntary detrusor contractions or reduced compliance during filling, a voiding pattern of low pressure and low flow, and urinary retention, as observed in urodynamic studies. However, among the elderly, DHIC entails pathological traits linked to both UAB and OAB [8]. Our previous data revealed overactive bladders induced by OHD, in a rat animal model to a human clinical trial [66]. The ovariectomized Sprague–Dawley rat model mimicking the physiological conditions of menopause for 12 months was utilized to induce OAB and assess the potential therapeutic mechanism of LiESWT (0.12 mJ/mm^2^, 300 pulses, and 3 pulses/s). Moreover, the randomized, single-blinded clinical trial enrolled 58 participants to investigate the therapeutic efficacy of LiESWT (0.25 mJ/mm^2^, 3000 pulses, 3 pulses/s) on postmenopausal women with OAB. In the human clinical trials, participants exhibited OAB symptoms, including decreased urinary frequency, nocturia, urgency, urgency incontinence, and PVR but increased voided urine volume and the maximal flow rate. The results reveal that 8 weeks of LiESWT attenuated the inflammatory responses, increased angiogenesis, and promoted proliferation and differentiation, thereby improving OAB symptom and, consequently, promoting social activity. 

In rat animal model, the bladder function was assessed through urodynamic parameters and voiding behavior, including peak micturition pressure, micturition frequency, voided volume, and nonvoided contraction. The OVX group revealed bladder hyperactivity, characterized by increased micturition frequency, peak micturition pressure, and nonvoiding contraction compared with the sham group. Conversely, both the OVX + SW4 group and the OVX + SW8 group demonstrated significant reductions in peak micturition pressure and micturition frequency, along with augmented bladder capacity compared with the OVX group. However, approximately one-third of the ovariectomized mice for 12 months showed no significant differences in bladder micturition pressures from the control group, and only showed symptoms such as increased urination frequency, nonvoiding contraction, and decreased urinary volume, which is an atypical overactive bladder. In this study, the CMG data on the sham group illustrate a consistent and steady micturation pattern, whereas the OVX group revealed bladder hyperactivity, characterized by an elevated micturition frequency, nonvoiding contractions, and decreased micturition volume. On the contrary, both the OVX + SW4 group and the OVX + SW8 group exhibited notable reductions in frequency and heightened micturition volume compared with the OVX group. The LiESWT can be applied as a potential therapeutic method for OAB and DHIC in clinical practice. 

Previous studies showed that the neuroprotective benefits of 17β-estradiol (E2) ameliorated cholinergic deficit, elevated the expression levels of choline acetyltransferase and 5-hydroxytryptamine receptor 2A, and lowered the expression of GFAP in a rat model of Alzheimer’s disease induced by OVX [77]. Specifically, E2 and progesterone promoted neuronal survival by protecting neurons following brain injury [78]. Therefore, ovarian hormones were potent regulators of neuronal cell survival in the central nervous system [79]. Moreover, the effects of E2 activation, via estrogen-receptor signaling and TRPV1 inhibition by capsaicin in rat nociception neurons, modulated bladder pain [21], suggesting that estrogen might reduce the excitatory effects of capsaicin and modulate pain to affect voiding function. The roles of TRP channels are to stabilize bladder contractile activity during the storage phase and regulate detrusor contractility and urothelial barrier function [80]. The influx of Ca^2+^ through different TRP channels induced cell depolarization in afferent nerve fibers and initiated the Ca^2+^-dependent signaling responses. A previous study also showed that postmenopausal women with OAB had relatively lower Ca^2+^ concentration [81]. The concentration of Ca^2+^ is essential for neuromuscular function and bladder contractility. The TRPV1 and TRPV4 of the capsaicin (vanilloid) receptor are the Ca^2+^ channels and are expressed within the lower urinary tract. Their presence extends beyond neuronal fibers to encompass urothelial, suburothelial, and muscular layers [24,82]. In response to the depletion of intracellular Ca^2+^ reservoirs, TRPV receptors may serve as facilitators for the influx of extracellular Ca^2+^ [38]. Applications of RTX in dorsal root ganglion cells lead to fragmentation of the ER, followed by the degradation of the plasma membrane and the formation of vesicles within the nuclear membrane, resulting in increased intracellular Ca^2+^ [83].

In animal studies, TRPV4 gene knockout mice exhibited abnormal micturition functions, including an increase in nonvoiding contractions [84]. Yoshiyama et al. also reported that TRPV4 gene knockout mice displayed OAB symptoms by voiding behavior in metabolic cage experiments [80]. These results indicate that TRPV4 has a crucial role in regulating detrusor contractility. Grundy et al. found that TRPV1 exhibited an augmenting effect on the afferent response to activation of P2X receptors within the mouse urinary bladder [85]. Activation of TRPV4 receptors using an agonist was found to have a notable impact. This included a reduction in pro-inflammatory chemokines and a reversal of macrophage phenotypic alterations. As a result, this intervention led to an amelioration of painful bladder hypersensitivity [86]. Masaru et al. found that a TRPV4 agonist improved bladder contractile function and pathological changes in lipopolysaccharide-induced painful bladder hypersensitivity. Neurogenic inflammation triggered the elevated expression of TRPV1 receptors within bladder suburothelium and c-fos protein in the dorsal root ganglia [87]. Clinically, an alleviation of sensory symptoms was observed subsequent to the utilization of RTX and capsaicin treatment for LUTS [85]. The overexpression of TRPV1 [25] and P2X3 receptors [26] in urothelium was shown in DO patients with OAB symptoms. As previously mentioned, the neuronal TRPV1 channel potentially plays a pathophysiological role in contributing to OAB symptoms and bladder pain [88]. Bladder sensory nerve fibers feature the presence of both TRPV1 and P2X receptors, which are associated with mechanosensation during the process of bladder filling.

There was a Ca^2+^-dependent process that could modulate the muscle contraction by a negative feedback manner [89], for example, the roles of voltage-dependent Ca^2+^ channels (VDCC), RyRs, large-conductance Ca^2+^-activated K^+^ (BK) channels, and small-conductance Ca^2+^-activated K^+^ (SK) channels in regulating the phasic contractions of guinea pig urinary bladder smooth muscle. RyRs have a significant role as negative feedback regulators of both contraction frequency and duration. This regulatory function is influenced by the activity of SK channels [89]. In addition, Ca^2+^ could influx across muscle sarcolemma through TRPV4, activate SK channels, and induce smooth muscle relaxation to prevent bladder hyperactivity [90]. Moreover, the anoctamin (ANO) family of calcium-activated chloride channels holds a range of diverse cellular functions, encompassing processes such as cell proliferation, survival, migration, contraction, and neuronal excitation. The acetylcholine (ACh)-induced contraction of the bladder’s detrusor muscle was inhibited through the use of the IP3 receptor antagonist heparin. Heparin acted by blocking the interaction between IP3 and its corresponding receptor, thereby preventing the release of IP3-sensitive intracellular Ca^2+^ [91,92]. In a rat model of metabolic syndrome, a Ca^2+^-induced Ca^2+^ release (CICR) response influenced the smooth muscle contraction in coronary arteries [93]. Impaired Ca^2+^ entry and RyR and SERCA modulation response damages could decrease the CICR response in smooth muscle, which might play a crucial role in OAB. In the present investigation, bladder detrusor contractile response using muscle strips was determined for synaptic transmission, receptor response, and smooth muscle contraction. The OHD status after 12 months of OVX had a worse bladder contractile response, which induced neuronal degeneration, and exhibited a meaningful reduction in the expressions of the TRPV channels (TRPV1 and TRPV4), ER-related Ca^2+^ channels/receptors (RyRs, IP3Rs, and SERCA), and Ano1, which resulted in being unable to trigger CICR and maintain the intracellular Ca^2+^ concentration (Figure 5 and Figure 6). In contrast, the therapeutic efficacy of the LiESWT improved bladder contractile function and enhanced neurogenesis and channel/receptor response to ameliorate detrusor hyperactivity. In addition, the levels of the serum calcium and phosphate concentrations were reduced in the OVX group, whereas the levels in the OVX + SW4 group and the OVX + SW8 group were similar to the sham group. However, there were no differences in the serum Ca/P ratios among the different groups, suggesting that serum Ca/P ratio is not a valuable tool in the diagnosis of OHD-induced DHIC.

There are ICs in bladder tissues which could participate in modulating the neurotransmission at nerve endings and smooth muscles. Recent studies have found that several types of ICs could regulate bladder activity by regulating the Ca^2+^ concentration [42,43,44] and transducing signals between urothelial and muscular layers [94]. Koh et al. found some ICs stained with PDGFRα had the purinergic relaxation effect and might participate in the regulation of the detrusor muscle [95,96]. Sanders et al. described that ICs could be a pacemaker region in the gastrointestinal tract [97] located between nerve endings and smooth muscle cells [98]. A previous study also revealed that the TRPV4 agonist could activate SK channels in PDGFRα^+^ ICs, cause Ca^2+^ influx through TRPV4 without initiating intracellular Ca^2+^ signaling, and, finally, result in the relaxation of bladder detrusor muscle strips [90]. Bladder ICs are also implicated in the underlying mechanisms of OAB pathophysiology. The exogenous stem cell factors derived from both neural and smooth muscle origins led to the restoration of detrusor contraction in rats with UAB. This improvement was achieved by augmenting the population of ICCs, thereby contributing to the enhancement of bladder function [99]. In a human clinical pilot study, the effectiveness and safety of intradetrusor injections of autologous muscle-derived cells were reported as a treatment for UAB [100]. In the present study, noteworthy reductions in the expressions of IC markers (C-kit, vimentin, and PDGFR) were observed in the OVX rats and was also found in comparison with the sham group, while the LiESWT enhanced the expressions of Ki67 and IC markers in the SL.

Our findings suggest that a decrease in ICs after OHD status could be a possible reason for DHIC, while LiESWT could promote IC regeneration and contribute to an improvement in detrusor hyperactivity symptoms. The dominant effect of LiESWT is thought to be the conversion of mechanotransduction into biochemical signals. Specific cellular processes or molecules for cellular signaling transduction modulated by LiESWT include ATP, P2X7 [101], extracellular-signal-regulated kinase (ERK) [102], protein kinase R-like ER kinase/activated transcription factor (PERK/ATF) [103], vascular endothelial growth factor and brain-derived neurotrophic factor (VEGF), and brain-derived neurotrophic factor (BDNF) [104]. A stretch-activated channel by LiESWT allowed for an influx of calcium to play a role in mechanotransduction. Wang et al. reported that 4 weeks of LiESWT (0.02 mJ/mm^2^ and 400 pulses) applied to streptozotocin-induced diabetic rats ameliorated the symptoms of DU with impaired contractility and urinary incontinence. Moreover, the LiESWT could increase bladder nerve innervation, improve bladder wall composition, activate bladder muscle regeneration, and enhance muscle contractile function [69]. In addition, 4 weeks of LiESWT (2000 shocks with a frequency of 3 Hz, at an energy level of 0.25 mJ/mm^2^ weekly) on the suprapubic bladder area improved urinary frequency, reduced inflammation, and led to a significant decrease in the Visual Analog Scale for Pain (VAS pain) in treating refractory IC/BPS [65]. Our previous study also showed that an 8-week regimen of LiESWT (0.25 mJ/mm^2^, 3000 pulses, and 3 Hz) could alleviate symptoms associated with OAB. This treatment exhibited the ability to enhance various urodynamic metrics, encompassing voided urine volume, Qmax, PVR, and functional bladder capacity [66]. In a study by Seo et al., increased levels of GFAP and NF200 were observed in LiESWT-treated rats with sciatic nerve injury, indicating nerve regeneration effects [105]. Zhang et al. further found that LiESWT has the potential to augment the proliferation and differentiation of neural stem cells. This modulation occurs via the activation of signaling pathways, including Notch, PI3K/AKT, and Wnt/β-catenin [101]. A previous study in a cat model also suggested that the contraction of the detrusor muscle in response to ACh is facilitated through the involvement of M3 muscarinic receptors, which trigger the activation of Gq/11 and phospholipase C-β1. This activation subsequently leads to the release of IP3-dependent Ca^2+^ from intracellular stores [106]. In the present study, LiESWT ameliorated bladder hyperactivity by improving urodynamic parameters and voiding behavior, encompassing micturition frequency, voided volume, and nonvoided contraction. Moreover, LiESWT treatment ameliorated bladder detrusor contractile response. Additionally, the neurogenesis effect of LiESWT increased the levels of neurogenesis (NF, NeuN, and GFAP), muscarinic receptors (M2 and M3), purinergic receptor (P2X7), TRPV channels (TRPV1 and TRPV4), ER stress proteins (CHOP, GRP78, and caspase 12), ER-related Ca^2+^ channels/receptors (RyR, IP3R, and SERCA) and Ano1. Meanwhile, LiESWT treatment significantly enhanced the signaling-pathway-related proteins, including Gq/11, Gq/12, Gq/13, RhoA, and RhoK, in the bladder involving the activation of ER-related Ca^2+^ channels/receptors (Figure 8). Therefore, the OVX rats had worse bladder contractile responses, which caused bladder contractile deficiency, while the LiESWT reduced the detrusor hyperactivity and ameliorated the bladder contractile function.

### Strengths and Limitations

In this study, muscle strips from OVX rats demonstrated lower contraction power when stimulated with EFS, carbachol, and KCl. These findings demonstrate the instability of the bladder and low intravesical pressure, which are similar to those observed in human DHIC. However, urodynamic study is still an essential tool for definitively diagnosing DHIC. It can reveal involuntary detrusor contractions or reduced compliance during filling, along with a relatively lower pressure, lower flow voiding pattern, and urinary retention. In addition, we could not detect the amount of PVR retained in the bladder after involuntary detrusor contractions using urodynamics study as a diagnostic tool in the rat model. Additionally, the study also had limitations regarding the duration and depth of exploration into the effects of the LiESWT on OHD. Specifically, it only examined the effects at W4 and W8, without delving into longer-term impacts, raising questions concerning sustainability and durability. Furthermore, while potential mechanisms underlying the therapeutic effects of the LiESWT on OHD were explored, the precise molecular pathways and cellular mechanisms involved may not be fully elucidated. We also did not evaluate the therapeutic efficacy of the LiESWT on the enzyme choline acetyltransferase of the bladder in the cholinergic neuronal cell activity for detrusor muscle contractility. In addition, intracellular and extracellular Ca^2+^ concentrations in bladder tissue for bladder hyperactivity in OVX-induced DHIC rats need to be further explored. 

We will explore the calcium concentration between the bladder tissue and serum for detrusor muscle contraction involved in regulating intracellular Ca^2+^ oscillation in a rat model of OHD-induced DHIC. In the future, using pharmacological agonists and antagonists of calcium channels (TRPV1, TRPV4, Ano1, and VDCC), ER-related Ca^2+^ channels/receptors (RyRs, IP3Rs, SERCA, and Ano1), purinergic (P2X3, P2X5, and P2X7), and muscarinic (M2 and M3) receptors in the muscle contractile experiments, we will investigate whether LiESWT modulates the generation of Ca^2+^ oscillations through activation of RyRs and IP3Rs in the ER membrane and Ano1 in the plasma membrane involved in the calcium signaling pathway.

## 4. Materials and Methods

### 4.1. Animals and OVX

This experimental procedure was granted approval by the Committee for the Use of Experimental Animal of Kaohsiung Medical University (IACUC: KMUH-110187; 109219). Thirty-two female Sprague–Dawley rats (purchased from the animal center of BioLASCO Taiwan Co., Ltd., Taipei, Taiwan), weighing between 200 and 250 g, were divided into four groups, including (A) the sham group, (B) the OVX group (OVX-induced ovarian hormone deficiency (OHD) for 12 months), (C) the OVX + SW4 group (OHD status for 12 months, followed by once weekly LiESWT for 4 weeks), and (D) the OVX + SW8 group (OHD status for 12 months, followed by twice weekly LiESWT for 4 weeks). OVX was performed under halothane anesthesia by a single surgeon. Bilateral ovaries were excised through incisions on both sides of the spine, each about 1 cm in length. The rats were placed in individual cages at room temperature with a 12-h light–dark cycle, and with free access to food and water during the entire experiment. Physical indicators, including water consumption, urine output, and body weight, were recorded after OHD status for 12 months. Micturition patterns by physical metabolic cage and cystometry data were collected to identify the symptoms of detrusor hyperactivity after 12 months of OVX.

### 4.2. LiESWT Treatment

**Figure 9:**
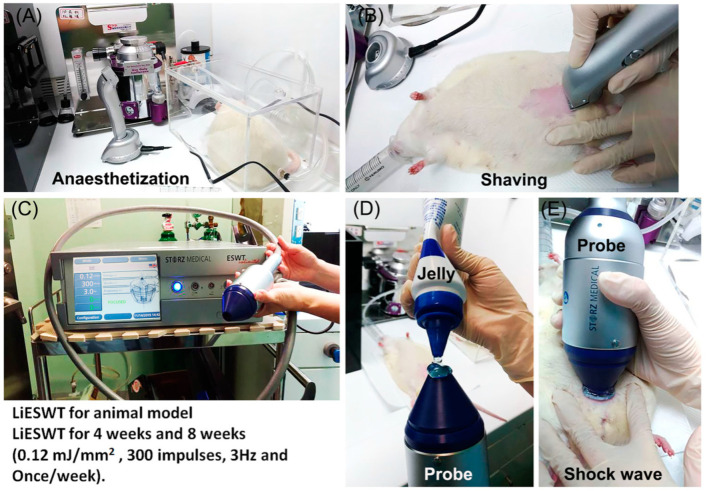
Images of the LiESWT applications in a rat model of OVX-induced detrusor hyperactivity with impaired contractility. The rats were anesthetized with (**A**) isoflurane and the (**B**) abdominal skin was shaved. (**C**) The LiESWT was performed using the DUOLITH SD1-TOP-focused shock wave system with an energy intensity of 0.12 mJ/mm^2^, frequency of 3 Hz, and 300 impulse shock waves. (**D**,**E**) The applicator was placed on the bladder area and covered with ultrasound transmission gel. LiESWT, low-intensity extracorporeal shock wave therapy.

The rats were anesthetized by isoflurane (Figure 9A), and their abdominal skins were shaved (Figure 9B). The LiESWT was performed by a DUOLITH SD1-TOP-focused shock wave system (STORZ MEDICAL, AG, Kreuzlingen, Switzerland). The energy of the LiESWT was set at an intensity of 0.12 mJ/mm^2^, a frequency of 3 Hz, and to 300 impulse shock waves (Figure 9C). The applicator was then placed on the skin of the bladder area with ultrasound transmission gel (Figure 9D,E).

### 4.3. Estradiol Level Measurements

Four weeks after OVX surgery, the serum estradiol level was checked. Blood was drawn from the tail vein under anesthesia and then centrifuged at 4 °C. Based on the 17β-estradiol ELISA kit (Cayman Chemical Co., Ann Arbor, MI, USA), the manufacturer’s protocol was followed. Microtiter wells from the kit were coated with a primary antibody that targeted the antigenic site of the estradiol molecule. Substrate solution (100 L) was added to each well of the serum samples and allowed to incubate for 15–20 min at room temperature. Then, 50 μL stop solution was added to determine the optical density of each well. The concentration of estradiol was measured from an ELISA reader (Bio-Tek ELX 800, BioTek, Bad, Germany). The average absorbance values of both the standard and experimental serum samples were calculated for various groups.

### 4.4. Measurements of Serum Calcium and Phosphate Levels after Treatment

Ultraviolet-visible spectroscopy was used to measure the levels of the serum calcium (Ca) and phosphate (P) concentrations. The ratio of the serum calcium conc. (mg/dL) to serum phosphate conc. (mg/dL) was analyzed. Serum calcium levels were categorized into 3 groups: low (<8.4 mg/dL), medium (≥8.4 < 10.0 mg/dL), and high (≥10.0 mg/dL). Additionally, serum phosphate levels were categorized into 3 groups: low (<3.5 mg/dL), medium (≥3.5–<6.0 mg/dL), and high (≥6.0 mg/dL).

### 4.5. CMG for Bladder Contraction

The rats were anesthetized by Zoletil-50 (1 mg/Kg, intraperitoneal injection (IP)). After analgesia, a PE50 tube was placed via the urethra to the bladder to empty it. A total of 0.9% normal saline was directed into the bladder via the urethral catheter at a steady rate (0.08 mL/min). The tube was connected to a pressure transducer to measure the intravesical pressure. After at least 5 cycles of the filling and voiding phases, the bladder entered into a steady phase. The signals were amplified (by ML866, PowerLab, ADInstrument) and recorded (by Labchart 7, ADInstruments: Windows 7 system). The CMG parameters included the filling pressure, peak micturition pressure, micturition volume, and nonvoiding contractions (bladder contracture without leakage of urine).

### 4.6. Measurements of the Micturition Volume and Frequency by Physiological Metabolic Cage

The micturition pattern was measured by individually placing rats from various groups into separate KDS-TL380 metabolic cages (R-2100; Lab Products, Rockville, MD, USA). The data were collected by an MLT0380 transducer (MLT 0380, ADI Instruments, Colorado Springs, CO, USA), and it recorded and analyzed the volumes of water consumed and urine output for 3 days.

### 4.7. Studies of Bladder Muscle Strips for Bladder Contractility

The muscle strip contractility was measured by the stimulation of the EFS. Bladder longitudinal strips (about 5 × 15 mm^2^) were obtained from the bladder trigone to dome. The strips were placed in oxygenated Krebs–Henseleit solution w a temperature of 37 °C for 30 min. An initial resting tension of 2 g was applied for 30 min. The strips were stimulated by electrical field at 2, 8, and 32 Hz, followed by carbachol (20 μM) and KCl (120 mM). The collected data were digitized and, subsequently, analyzed using the Grass POLYVIEW A-D & conversion system (Grass Instrument Co, Warwick, RI, USA).

### 4.8. Masson’s Trichrome Staining for Morphological Change

To investigate pathological alterations within the bladder, Masson’s trichrome stain was employed. Following fixation in 4% paraformaldehyde for a minimum of 24 h at 4 °C, bladder tissue samples underwent paraffin embedding. Subsequently, sections of bladder tissue measuring 5 µm in thickness were prepared for Masson’s trichrome staining using the Masson’s Trichrome Stain Kit (Sigma, HT15, St. Louis, MO, USA). This staining technique facilitated the examination of bladder pathomorphology. Standard Masson’s trichrome staining protocol was applied, which resulted in connective tissue being labeled in blue and DSM in red. The bladder tissue slides, stained using Masson’s Trichrome staining, were subjected to evaluation by two independent pathologists for a comprehensive analysis.

### 4.9. Western Blot Analysis for Protein Expression

The bladder was separated into mucosa and muscle layers. Frozen bladder tissue samples were homogenized using lysis buffer (50 mM Tris, pH 7.5, 5% Triton-X100) with Halt Protease Inhibitor Cocktail (Pierce, Rockford, IL, USA) on ice and centrifuged at 14,000× *g* at 4 °C for 20 min. Equal quantities of total protein (20 μg) were separated on 12% SDS polyacrylamide gels and then transferred onto PVDF membranes. After being blocked with 5% nonfat milk, the membranes used for blotting were, subsequently, exposed to the primary antibody, including neurogenesis-related markers [neurofilament, NeuN, GFAP, muscarinic receptors (M2 and M3), and purinergic receptor (P2X7)], angiogenesis markers (α-SMA, laminin, and integrin-α6), interstitial markers (C-Kit, vimentin, and PDGFR), transient potential vanilloid channels (TRPV1 and TRPV4), ER-stress-related proteins (GRP78, CHOP, and caspase 12) and calcium channels (RyR, IP3R, SERCA, and Ano1), and cell-signal-related proteins (Gα 11, Gα 12, Gα 13, RhoA, and RhoK). The obtained results were standardized using glyceraldehyde-3-phosphate dehydrogenase (GAPDH; Merck, mouse monoclonal IgG, 1:2,500, MW: 36 kDa, catalog no. MAB374) as a reference. The blots were visualized using enhanced chemiluminescence (ECL) and then exposed to Biomax L film (Kodak). In each experiment, a negative control was included in which no primary antibody was applied. Each Western blotting procedure was replicated three times, and the resulting blots were analyzed using ImageJ software. Additional materials and methods utilized in the Western blot experiments are outlined in detail in Appendix A.

### 4.10. Immunofluorescence Staining to Spot the Location of Protein Expression

The bladder tissues were fixed in 4% paraformaldehyde in PBS (0.1 M, pH 7.4 phosphate-buffered saline) overnight. Subsequently, the tissues were embedded in paraffin and sliced into sections with a thickness of 5 µm. Double-immunofluorescence staining was executed to determine the specific localization of the target protein, following methods previously published in the literature [74,75]. The bladder sections underwent blocking with 10% NGS in PBS/0.5% Triton X-100 for a duration of 1 h. Subsequently, the sections were subjected to incubation with primary antibodies directed toward M2 (Abcam, mouse monoclonal antibody, 1:100), NF (Novus, mouse monoclonal antibody, 1:100, catalog no. NB500-416), TRPV4 (Affinity biosciences, rabbit polyclonal antibody, 1:100, catalog no. DF8624), IP3R (Abcam, mouse monoclonal antibody, 1:100, catalog no. ab255762), GRP78 (Proteintech, rabbit polyclonal antibody, 1:100, catalog no. 11587-1-AP), SERCA (Santa Cruz Biotechnology, mouse monoclonal antibody, 1:100, catalog no. sc-376235), and α-SMA (Abcam, rabbit polyclonal antibody, 1:100, catalog no. ab5694) at a temperature of 4 °C overnight. Tissues were then rinsed with PBS/0.5% Triton X-100 for a duration of 15 min, followed by incubation with secondary antibodies (1:800; Invitrogen) at room temperature for 1 h. After washing the tissues with PBS buffer, DAPI was applied, and the samples were cover-slipped using Prolong Gold anti-fade reagent (Invitrogen). Additionally, a negative control was conducted to distinguish nonspecific immunostaining by omitting the primary antibody.

### 4.11. Statistical Analysis

An analysis of the variance with the Bonferroni test was computed, followed by a two-way analysis of the variance to compare the differences among the various groups. Following three independent experimental repetitions, the mean values, standard deviations (SDs), and *p*-values were subjected to analysis. The *p*-values were determined through a Student’s *t*-test, and a result was deemed significant when the *p*-value was less than 0.05.

## 5. Conclusions

The OHD status after 12 months of OVX had a worse bladder contractile response, induced neuronal degeneration, and exhibited meaningful reductions in TRPV channels (TRPV1 and TRPV 4), ER-related Ca^2+^ channels/receptors (RyR, IP3R, and SERCA), and Ano1 involved in regulating intracellular Ca^2+^ oscillation. However, the therapeutic effect of the LiESWT improved bladder contractile function and enhanced neurogenesis and channel/receptor responses to alleviate detrusor hyperactivity in a rat model of OHD-induced DHIC. LiESWT may be applied as a potential therapeutic method for OAB and DHIC in future clinical practice.

## Figures and Tables

**Table 1:** Experimental design.

Animal model Thirty-two female Sprague-Dawley rats were divided into four groups, including (A) The sham group; (B) The OVX group: OVX-induced OHD for 12 months; (C) The OVX + SW4 group: OHD status for 12 months, followed by once weekly LiESWT for 4 weeks. The energy of LiESWT was set at the intensity of 0.12 mJ/mm^2^, the frequency of 3 Hz and 300 impulse shock waves; (D) The OVX + SW8 group: OHD status for 12 months, followed by twice weekly LiESWT for 4 weeks.
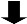
Materials and Methods Serum parameters, physical indicators and urodynamic parameters for the different groups. (Table 2) Urodynamic study parameters and voiding behavior for bladder function: Cystometrogram and micturition patterns by physical metabolic cage. (Figure 1) Bladder strip preparation for contractile responses of detrusor muscle: Electrical field stimulation (EFS: 2, 8, and 32 Hz), carbachol (20 μM), and KCl (120 mM). The contractile response of the bladder detrusor was evaluated in terms of synaptic transmission, receptor activity and muscle contraction (Figure 2). Mason’s Trichrome staining for bladder pathologic alteration (Figure 3). Western blotting and immunofluorescence analysis for marker protein expression (1) Myofibroblastic cell phenotype analysis: Ki67, α-SMA, laminin and vimentin (Figure 3). (2) Bladder angiogenic remodeling: Angiogenesis markers (α-SMA, laminin and integrin-α6) (Figure 3). (3) Interstitial cell generation: Interstitial cell markers (C-Kit, vimentin and PDGFR) (Figure 3). (4) Bladder neurogenesis, including neuronal regeneration, synaptic transmission and receptor response: Neurogenesis-related markers (NF, NeuN, GFAP, muscarinic receptor (M2 and M3) and purinergic receptor (P2X7)) (Figure 4). (5) TRPV channel for nociception and mechanosensory transduction to modulated the calcium concentration: TRPV markers (TRPV1 and TRPV4) (Figure 5). (6) ER stress protein (CHOP, GRP78 and Caspase 12) and Ano1 for detrusor muscle contractility (Figure 6). (7) Cell-signal-related proteins involved in the activation of ER-related Ca^2+^ channels/receptors to modulate the generation of Ca^2+^ oscillation: Gq/11, Gq/12, Gq/13, RhoA and RhoK (Figure 7).

Note: α-SMA, α-smooth muscle actin; Ano1, anoctamin-1; CHOP, C/EBP homologous protein; ER, endoplasmic reticulum; GRP78, Glucose-regulated protein 78; LiESWT, low-intensity extracorporeal shock wave therapy; NeuN, neuronal nuclei; NF, neurofilament; OHD, ovarian hormone deficiency; TRPV, transient receptor potential vanilloid. RhoA, ras homolog family member A; RhoK, Rho-associated protein kinase.

**Table 2:** Serum parameters, physical indicators, and urodynamic parameters for the different groups.

Groups	Sham	OVX	OVX + SW4	OVX + SW8
No. rats	8	8	8	8
Serum parameters				
Serum estradiol concentration (pg/mL) before treatment	32.3 ± 1.3	32.4 ± 1.4	30.0 ± 1.4	31.8 ± 1.2
Serum estradiol concentration (pg/mL) after treatment	33.5 ± 3.4	16.4 ± 1.3 **	15.6 ± 1.0 **	15.5 ± 1.4 **
Serum calcium concentration (mg/dL) after treatment	10.5 ± 0.3	9.4 ± 0.4 *	10.2 ± 0.5 ^#^	10.5 ± 0.3 ^#^
Serum phosphate concentration (mg/dL) after treatment	5.3 ± 0.8	3.7 ± 0.7 *	4.7 ± 1.0	4.3 ± 0.6
The ratio of serum calcium concentration (mg/dL)/serum phosphate concentration (mg/dL)	2.1 ± 0.4	2.5 ± 0.6	2.2 ± 0.4	2.4 ± 0.2
Physical indicators				
Water intake (mL/24 h)	43.2 ± 15.1	36.4 ± 5.5	36.7 ± 4.8	34.6 ± 6.8
Urine output (mL/24 h)	22.2 ± 2.8	15.5 ± 5.4	18.2 ± 6.3	19.1 ± 6.8
Body weight (g)	406.8 ± 46.8	569.2 ± 79.2 **	518.8 ± 61.0 **	528.2 ± 57.7 **
Bladder weight (mg)	169.2 ± 18.8	173.2 ± 16.1	169.6 ± 39.9	181.4 ± 27.8
Ratio of bladder weight (mg)/body weight (g)	0.42 ± 0.06	0.30 ± 0.05 **	0.32 ± 0.08 *	0.34 ± 0.10 *
Urodynamic parameters				
Frequency (No. voids/1 h)	4.8 ± 0.9	9.8 ± 2.1 **	5.9 ± 1.1 *^,##^	4.9 ± 1.0 ^##,†^
Peak micturition pressure (cm H_2_O)	26.0 ± 2.5	28.6 ± 5.2	27.8 ± 6.5	25.8 ± 3.6
Voided volume (mL)	2.9 ± 0.6	1.4 ± 0.3 **	2.4 ± 0.7 ^#^	2.9 ± 0.7 ^##^
No. of nonvoiding contractions between micturitions (No. voids/h)	0.00 ± 0.00	4.63 ± 0.90 **	2.20 ± 0.30 *^,##^	0.30 ± 0.07 *^,##,†^

OVX, bilateral ovariectomy; OVX + SW4, OHD status for 12 months, followed by once weekly LiESWT for 4 weeks; OVX + SW8, OHD status for 12 months, followed by twice weekly LiESWT for 4 weeks. LiESWT, low-intensity extracorporeal shockwave therapy. Values are the means ± SD. * *p* < 0.05 and ** *p* < 0.01 versus the sham group; ^#^ *p* < 0.05 and ^##^ *p* < 0.01 versus the OVX group; ^†^ *p* < 0.05 versus the OVX + SW4 group.

## Data Availability

Dataset available on request from the authors.

## Abbreviations

α-SMA, α-smooth muscle actin; Ano1, anoctamin-1; BOO, bladder outlet obstruction; CHOP, C/EBP homologous protein; CICR, Ca^2+^-induced Ca^2+^ release; CMG, cystometrogram; CP/CPPS, chronic prostatitis/chronic pelvic pain syndrome; DHIC, detrusor hyperactivity with impaired contractility; DO, detrusor overactivity; DU, detrusor underactivity; ED, erectile dysfunction; ER, endoplasmic reticulum; GFAP, glial fibrillary acidic protein; GRP 78, glucose-regulated protein 78; ICs, interstitial cells; ICCs, interstitial Cajal cells; ICLCs, interstitial Cajal-like cells; IC/BPS, interstitial cystitis and bladder pain syndrome; IP3Rs, inositol triphosphate receptors; LiESWT, low-intensity extracorporeal shock wave therapy; LUTS, lower urinary tract symptoms; NeuN, neuronal nuclei; NF, neurofilament; OAB, overactive bladder; OHD, ovarian hormone deficiency; OVX, bilateral ovariectomy; PVR, postvoid residual volume; Qmax, maximum urinary flow rate; RTX, resiniferatoxin; RyRs, ryanodine receptors; SERCA, sarco/endoplasmic reticulum Ca^2+^-ATPase; SR, sarcoplasmic reticulum; SUI, stress urinary incontinence; TRPV1, transient receptor potential vanilloid 1; TRPA1, transient receptor potential ankyrin type 1; TRPM8, transient receptor potential melastatin type 8; UAB, underactive bladder; UUI, urgency urinary incontinence; VDCC, voltage-dependent Ca^2+^ channels.

## Appendix A. Other Materials and Procedures Used in the Western Blot Experiments

The membranes were incubated with the indicated primary antibodies as follows: neurofilament (NF; Novus, mouse monoclonal, 1:1000, MW: 210 kDa, catalog no. NB500-416), NeuN (Merck, mouse monoclonal antibody, 1:1000, MK: 44-48 kDa, catalog no. #MAB377), GFAP (Affinity biosciences, mouse monoclonal antibody, 1:2000, MW: 50 kDa, catalog no. BF0345), M2 (Abcam, mouse monoclonal antibody, 1:1000, MK: 52 kDa, catalog no. ab2805), M3 (Abcam, rabbit polyclonal antibody, 1:1000, MW: 85 kDa, catalog no. ab87199), P2X7 (Abcam, rabbit polyclonal antibody, 1:1000, MW: 68 kDa, catalog no. ab109054), TRPV1 (LSBio, rabbit polyclonal antibody, 1:1000, MW: 100 kDa, catalog no. LS-C827828), TRPV4 (Affinity biosciences, rabbit polyclonal antibody, 1:1000, MW: 98 kDa, catalog no. DF8624), Ki67 (Abcam, rabbit monoclonal IgG 1:1000, MW: 358 kDa, catalog no. ab1667), C-kit (Bioss, rabbit polyclonal antibody, 1:1000, MW: 120, 140 kDa, catalog no. bs-10005R), vimentin (R&D System, mouse monoclonal antibody, 1:1000, MK: 55 kDa, catalog no. MAB21052), PDGFR (Abcam, rabbit monoclonal antibody, 1:1000, MK: 170 kDa, catalog no. ab215978), α-SMA (Abcam, rabbit polyclonal antibody, 1:6000, MK: 40, 42 kDa, catalog no. ab5694), laminin (Abcam, rabbit polyclonal antibody, 1:1000, MK: 200, 400 kDa, catalog no. ab7463), integrin-α6 (Laminin receptor; Abcam, rabbit monoclonal antibody, 1:5000, MK: 127 kDa, catalog no. ab181551), CHOP (Novus, mouse monoclonal antibody, 1:1000, MK: 19kDa, catalog no. NB600-1335), GRP78 (Proteintech, rabbit polyclonal antibody, 1:1000, MW: 78 kDa, catalog no. 11587-1-AP), caspase 12 (Abcam, rabbit polyclonal antibody, 1:1000, MK: 55 kDa, catalog no. ab18766), IP3Rs (Abcam, mouse monoclonal antibody, 1:1000, MK: 314 kDa, catalog no. ab255762), RyRs (Abcam, rabbit monoclonal antibody, 1:1000, MK: 565 kDa, catalog no. ab231086), SERCA (Santa Cruz, mouse monoclonal antibody, 1:1000, MW: 100 kDa, catalog no. sc-376235), Ano1 (Santa Cruz, mouse monoclonal antibody, 1:1000, MW: 114 kDa, catalog no. sc-377115), Gα 11 (Santa Cruz, mouse monoclonal antibody, 1:1000, MW: 45 kDa, catalog no. sc-390382), Gα 12 (Santa Cruz, mouse monoclonal antibody, 1:1000, MW: 45 kDa, catalog no. sc-515445), Gα 13 (Santa Cruz, mouse monoclonal antibody, 1:1000, MW: 44 kDa, catalog no. sc-293424), RhoA (Novus, mouse monoclonal antibody, 1:800, MK: 22 kDa, catalog no. NBP2-22528), RhoK (invitrogen, rabbit polyclonal antibody, MW: 70kDa, catalog no. PA5-115326), ß-actin (Cell Signaling, rabbit monoclonal IgG, 1:5,000, MW: 43 kDa, catalog no. 4970S), and glyceraldehyde-3-phosphate dehydrogenase (GAPDH; Merck, mouse monoclonal IgG, 1:2,500, MW: 36 kDa, catalog no. MAB374).

## References

[1] Syan R., Brucker B.M. (2016). Guideline of guidelines: Urinary incontinence. BJU Int..

[2] Kuo H.C. (2002). Analysis of the pathophysiology of lower urinary tract symptoms in patients after prostatectomy. Urol. Int..

[3] Kuo H.C. (2007). Videourodynamic analysis of pathophysiology of men with both storage and voiding lower urinary tract symptoms. Urology.

[4] Abarbanel J., Marcus E.L. (2007). Impaired detrusor contractility in community-dwelling elderly presenting with lower urinary tract symptoms. Urology.

[5] Ong H.L., Kuo H.C. (2019). Bladder dysfunction does not affect long-term success rate of the retropubic suburethral sling procedure in women with stress urinary incontinence. Low. Urin. Tract Symptoms.

[6] Li X., Liao L.M., Chen G.Q., Wang Z.X., Lu T.J., Deng H. (2018). Clinical and urodynamic characteristics of underactive bladder: Data analysis of 1726 cases from a single center. Medicine.

[7] Stav K., Shilo Y., Zisman A., Lindner A., Leibovici D. (2013). Comparison of lower urinary tract symptoms between women with detrusor overactivity and impaired contractility, and detrusor overactivity and preserved contractility. J. Urol..

[8] Chancellor M.B. (2014). The overactive bladder progression to underactive bladder hypothesis. Int. Urol. Nephrol..

[9] Yang T.H., Chuang F.C., Kuo H.C. (2018). Urodynamic characteristics of detrusor underactivity in women with voiding dysfunction. PLoS ONE.

[10] Lee C.L., Kuo H.C. (2019). Efficacy and safety of mirabegron, a beta(3)-adrenoceptor agonist, in patients with detrusor hyperactivity and impaired contractility. Low. Urin. Tract. Symptoms.

[11] Wang C.C., Lee C.L., Kuo H.C. (2016). Efficacy and Safety of Intravesical OnabotulinumtoxinA Injection in Patients with Detrusor Hyperactivity and Impaired Contractility. Toxins.

[12] Ciofu I., Ceausu I., Chirca N.M., Persu C. (2022). Solifenacin Treatment After Intradetrusor Injections With Botulinum Toxin in Patients With Neurogenic Detrusor Overactivity. Am. J. Ther..

[13] Hennessey D.B., Hoag N., Gani J. (2017). Sacral neuromodulation for detrusor hyperactivity with impaired contractility. Neurourol. Urodyn..

[14] Cheng C.L., de Groat W.C. (2014). Effects of agonists for estrogen receptor alpha and beta on ovariectomy-induced lower urinary tract dysfunction in the rat. Am. J. Physiol. Ren. Physiol..

[15] Imamov O., Yakimchuk K., Morani A., Schwend T., Wada-Hiraike O., Razumov S., Warner M., Gustafsson J.A. (2007). Estrogen receptor beta-deficient female mice develop a bladder phenotype resembling human interstitial cystitis. Proc. Natl. Acad. Sci. USA.

[16] Lee Y.L., Lin K.L., Wu B.N., Chuang S.M., Wu W.J., Lee Y.C., Ho W.T., Juan Y.S. (2018). Epigallocatechin-3-gallate alleviates bladder overactivity in a rat model with metabolic syndrome and ovarian hormone deficiency through mitochondria apoptosis pathways. Sci. Rep..

[17] Losordo D.W., Isner J.M. (2001). Estrogen and angiogenesis: A review. Arterioscler. Thromb. Vasc. Biol..

[18] Aikawa K., Sugino T., Matsumoto S., Chichester P., Whitbeck C., Levin R.M. (2003). The effect of ovariectomy and estradiol on rabbit bladder smooth muscle contraction and morphology. J. Urol..

[19] Papka R.E., Mowa C.N. (2003). Estrogen receptors in the spinal cord, sensory ganglia, and pelvic autonomic ganglia. Int. Rev. Cytol..

[20] Bennett H.L., Gustafsson J.A., Keast J.R. (2003). Estrogen receptor expression in lumbosacral dorsal root ganglion cells innervating the female rat urinary bladder. Auton. Neurosci..

[21] Xu S., Cheng Y., Keast J.R., Osborne P.B. (2008). 17beta-estradiol activates estrogen receptor beta-signalling and inhibits transient receptor potential vanilloid receptor 1 activation by capsaicin in adult rat nociceptor neurons. Endocrinology.

[22] Andersson K.E., Behr-Roussel D., Denys P., Giuliano F. (2022). Acute Intravesical Capsaicin for the Study of TRPV1 in the Lower Urinary Tract: Clinical Relevance and Potential for Innovation. Med. Sci..

[23] Vincent F., Duncton M.A. (2011). TRPV4 agonists and antagonists. Curr. Top. Med. Chem..

[24] Andersson K.E. (2019). TRP Channels as Lower Urinary Tract Sensory Targets. Med. Sci..

[25] Apostolidis A., Brady C.M., Yiangou Y., Davis J., Fowler C.J., Anand P. (2005). Capsaicin receptor TRPV1 in urothelium of neurogenic human bladders and effect of intravesical resiniferatoxin. Urology.

[26] Sun Y., Chai T.C. (2004). Up-regulation of P2X3 receptor during stretch of bladder urothelial cells from patients with interstitial cystitis. J. Urol..

[27] Zhang H.Y., Chu J.F., Li P., Li N., Lv Z.H. (2015). Expression and diagnosis of transient receptor potential vanilloid1 in urothelium of patients with overactive bladder. J. Biol. Regul. Homeost. Agents.

[28] Kim D.Y., Chancellor M.B. (2000). Intravesical neuromodulatory drugs: Capsaicin and resiniferatoxin to treat the overactive bladder. J. Endourol..

[29] Brady C.M., Apostolidis A., Yiangou Y., Baecker P.A., Ford A.P., Freeman A., Jacques T.S., Fowler C.J., Anand P. (2004). P2X3-immunoreactive nerve fibres in neurogenic detrusor overactivity and the effect of intravesical resiniferatoxin. Eur. Urol..

[30] Pandita R.K., Persson K., Andersson K.E. (1997). Capsaicin-induced bladder overactivity and nociceptive behaviour in conscious rats: Involvement of spinal nitric oxide. J. Auton. Nerv. Syst..

[31] Isogai A., Lee K., Mitsui R., Hashitani H. (2016). Functional coupling of TRPV4 channels and BK channels in regulating spontaneous contractions of the guinea pig urinary bladder. Pflug. Arch..

[32] Everaerts W., Zhen X., Ghosh D., Vriens J., Gevaert T., Gilbert J.P., Hayward N.J., McNamara C.R., Xue F., Moran M.M. (2010). Inhibition of the cation channel TRPV4 improves bladder function in mice and rats with cyclophosphamide-induced cystitis. Proc. Natl. Acad. Sci. USA.

[33] Cho K.J., Park E.Y., Kim H.S., Koh J.S., Kim J.C. (2014). Expression of transient receptor potential vanilloid 4 and effects of ruthenium red on detrusor overactivity associated with bladder outlet obstruction in rats. World J. Urol..

[34] Deruyver Y., Weyne E., Dewulf K., Rietjens R., Pinto S., Van Ranst N., Franken J., Vanneste M., Albersen M., Gevaert T. (2018). Intravesical Activation of the Cation Channel TRPV4 Improves Bladder Function in a Rat Model for Detrusor Underactivity. Eur. Urol..

[35] Merrill L., Gonzalez E.J., Girard B.M., Vizzard M.A. (2016). Receptors, channels, and signalling in the urothelial sensory system in the bladder. Nat. Rev. Urol..

[36] Caterina M.J., Schumacher M.A., Tominaga M., Rosen T.A., Levine J.D., Julius D. (1997). The capsaicin receptor: A heat-activated ion channel in the pain pathway. Nature.

[37] Sharma S.K., Vij A.S., Sharma M. (2013). Mechanisms and clinical uses of capsaicin. Eur. J. Pharmacol..

[38] Vangeel L., Voets T. (2019). Transient Receptor Potential Channels and Calcium Signaling. Cold Spring Harb. Perspect. Biol..

[39] Birder L.A., Kanai A.J., de Groat W.C., Kiss S., Nealen M.L., Burke N.E., Dineley K.E., Watkins S., Reynolds I.J., Caterina M.J. (2001). Vanilloid receptor expression suggests a sensory role for urinary bladder epithelial cells. Proc. Natl. Acad. Sci. USA.

[40] Voets T., Prenen J., Vriens J., Watanabe H., Janssens A., Wissenbach U., Bodding M., Droogmans G., Nilius B. (2002). Molecular determinants of permeation through the cation channel TRPV4. J. Biol. Chem..

[41] Jones J.L., Peana D., Veteto A.B., Lambert M.D., Nourian Z., Karasseva N.G., Hill M.A., Lindman B.R., Baines C.P., Krenz M. (2019). TRPV4 increases cardiomyocyte calcium cycling and contractility yet contributes to damage in the aged heart following hypoosmotic stress. Cardiovasc. Res..

[42] Kubota Y., Biers S.M., Kohri K., Brading A.F. (2006). Effects of imatinib mesylate (Glivec) as a c-kit tyrosine kinase inhibitor in the guinea-pig urinary bladder. Neurourol. Urodyn..

[43] Juszczak K., Maciukiewicz P., Drewa T., Thor P.J. (2014). Cajal-like interstitial cells as a novel target in detrusor overactivity treatment: True or myth?. Cent. Eur. J. Urol..

[44] Sanders K.M., Ward S.M., Koh S.D. (2014). Interstitial cells: Regulators of smooth muscle function. Physiol. Rev..

[45] Tokutomi N., Maeda H., Tokutomi Y., Sato D., Sugita M., Nishikawa S., Nishikawa S., Nakao J., Imamura T., Nishi K. (1995). Rhythmic Cl^−^ current and physiological roles of the intestinal c-kit-positive cells. Pflug. Arch..

[46] Huizinga J.D., Zhu Y., Ye J., Molleman A. (2002). High-conductance chloride channels generate pacemaker currents in interstitial cells of Cajal. Gastroenterology.

[47] Zhu Y., Mucci A., Huizinga J.D. (2005). Inwardly rectifying chloride channel activity in intestinal pacemaker cells. Am. J. Physiol. Gastrointest. Liver Physiol..

[48] Steiner C., Gevaert T., Ganzer R., De Ridder D., Neuhaus J. (2018). Comparative immunohistochemical characterization of interstitial cells in the urinary bladder of human, guinea pig and pig. Histochem. Cell Biol..

[49] Zhao M., Chen Z., Liu L., Ding N., Wen J., Liu J., Wang W., Ge N., Zu S., Song W. (2021). Functional Expression of Transient Receptor Potential and Piezo1 Channels in Cultured Interstitial Cells of Human-Bladder Lamina Propria. Front. Physiol..

[50] Foskett J.K., White C., Cheung K.H., Mak D.O. (2007). Inositol trisphosphate receptor Ca2+ release channels. Physiol. Rev..

[51] Meissner G. (1994). Ryanodine receptor/Ca^2+^ release channels and their regulation by endogenous effectors. Annu. Rev. Physiol..

[52] Martin-Cano F.E., Gomez-Pinilla P.J., Pozo M.J., Camello P.J. (2009). Spontaneous calcium oscillations in urinary bladder smooth muscle cells. J. Physiol. Pharmacol..

[53] Lee Y.C., Chuang S.M., Lin K.L., Chen W.C., Lu J.H., Chueh K.S., Shen M.C., Liu L.W., Long C.Y., Juan Y.S. (2020). Low-Intensity Extracorporeal Shock Wave Therapy Ameliorates the Overactive Bladder: A Prospective Pilot Study. Biomed. Res. Int..

[54] Long C.Y., Lin K.L., Lee Y.C., Chuang S.M., Lu J.H., Wu B.N., Chueh K.S., Ker C.R., Shen M.C., Juan Y.S. (2020). Therapeutic effects of Low intensity extracorporeal low energy shock wave therapy (LiESWT) on stress urinary incontinence. Sci. Rep..

[55] Zimmermann R., Cumpanas A., Miclea F., Janetschek G. (2009). Extracorporeal shock wave therapy for the treatment of chronic pelvic pain syndrome in males: A randomised, double-blind, placebo-controlled study. Eur. Urol..

[56] Moayednia A., Haghdani S., Khosrawi S., Yousefi E., Vahdatpour B. (2014). Long-term effect of extracorporeal shock wave therapy on the treatment of chronic pelvic pain syndrome due to non bacterial prostatitis. J. Res. Med. Sci..

[57] Guu S.J., Geng J.H., Chao I.T., Lin H.T., Lee Y.C., Juan Y.S., Liu C.C., Wang C.J., Tsai C.C. (2018). Efficacy of Low-Intensity Extracorporeal Shock Wave Therapy on Men With Chronic Pelvic Pain Syndrome Refractory to 3-As Therapy. Am. J. Mens. Health.

[58] Yuan P., Ma D., Zhang Y., Gao X., Liu Z., Li R., Wang T., Wang S., Liu J., Liu X. (2019). Efficacy of low-intensity extracorporeal shock wave therapy for the treatment of chronic prostatitis/chronic pelvic pain syndrome: A systematic review and meta-analysis. Neurourol. Urodyn..

[59] Guu S.J., Liu C.C., Juan Y.S., Li C.C., Tsai C.C. (2020). The 12-month follow-up of the low-intensity extracorporeal shockwave therapy in the treatment of patients with chronic pelvic pain syndrome refractory to 3-As medications. Aging Male.

[60] Chung E., Wang J. (2017). A state-of-art review of low intensity extracorporeal shock wave therapy and lithotripter machines for the treatment of erectile dysfunction. Expert. Rev. Med. Devices.

[61] Chung E., Cartmill R. (2015). Evaluation of clinical efficacy, safety and patient satisfaction rate after low-intensity extracorporeal shockwave therapy for the treatment of male erectile dysfunction: An Australian first open-label single-arm prospective clinical trial. BJU Int..

[62] Clavijo R.I., Kohn T.P., Kohn J.R., Ramasamy R. (2017). Effects of Low-Intensity Extracorporeal Shockwave Therapy on Erectile Dysfunction: A Systematic Review and Meta-Analysis. J. Sex. Med..

[63] Dong L., Chang D., Zhang X., Li J., Yang F., Tan K., Yang Y., Yong S., Yu X. (2019). Effect of Low-Intensity Extracorporeal Shock Wave on the Treatment of Erectile Dysfunction: A Systematic Review and Meta-Analysis. Am. J. Mens. Health.

[64] Sokolakis I., Hatzichristodoulou G. (2019). Clinical studies on low intensity extracorporeal shockwave therapy for erectile dysfunction: A systematic review and meta-analysis of randomised controlled trials. Int. J. Impot. Res..

[65] Chuang Y.C., Meng E., Chancellor M., Kuo H.C. (2020). Pain reduction realized with extracorporeal shock wave therapy for the treatment of symptoms associated with interstitial cystitis/bladder pain syndrome-A prospective, multicenter, randomized, double-blind, placebo-controlled study. Neurourol. Urodyn..

[66] Lin K.L., Lu J.H., Chueh K.S., Juan T.J., Wu B.N., Chuang S.M., Lee Y.C., Shen M.C., Long C.Y., Juan Y.S. (2021). Low-Intensity Extracorporeal Shock Wave Therapy Promotes Bladder Regeneration and Improves Overactive Bladder Induced by Ovarian Hormone Deficiency from Rat Animal Model to Human Clinical Trial. Int. J. Mol. Sci..

[67] Mykoniatis I., Kalyvianakis D., Zilotis F., Kapoteli P., Fournaraki A., Poulios E., Hatzichristou D. (2021). Evaluation of a low-intensity shockwave therapy for chronic prostatitis type IIIb/chronic pelvic pain syndrome: A double-blind randomized sham-controlled clinical trial. Prostate Cancer Prostatic Dis..

[68] Anothaisintawee T., Attia J., Nickel J.C., Thammakraisorn S., Numthavaj P., McEvoy M., Thakkinstian A. (2011). Management of chronic prostatitis/chronic pelvic pain syndrome: A systematic review and network meta-analysis. JAMA.

[69] Wang H.S., Oh B.S., Wang B., Ruan Y., Zhou J., Banie L., Lee Y.C., Tamaddon A., Zhou T., Wang G. (2018). Low-intensity extracorporeal shockwave therapy ameliorates diabetic underactive bladder in streptozotocin-induced diabetic rats. BJU Int..

[70] Wang H.J., Su C.H., Chen Y.M., Yu C.C., Chuang Y.C. (2022). Molecular Effects of Low-Intensity Shock Wave Therapy on L6 Dorsal Root Ganglion/Spinal Cord and Blood Oxygenation Level-Dependent (BOLD) Functional Magnetic Resonance Imaging (fMRI) Changes in Capsaicin-Induced Prostatitis Rat Models. Int. J. Mol. Sci..

[71] Wess O.J. (2008). A neural model for chronic pain and pain relief by extracorporeal shock wave treatment. Urol. Res..

[72] Hausdorf J., Lemmens M.A., Heck K.D., Grolms N., Korr H., Kertschanska S., Steinbusch H.W., Schmitz C., Maier M. (2008). Selective loss of unmyelinated nerve fibers after extracorporeal shockwave application to the musculoskeletal system. Neuroscience.

[73] Wang H.J., Tyagi P., Chen Y.M., Chancellor M.B., Chuang Y.C. (2019). Low Energy Shock Wave Therapy Inhibits Inflammatory Molecules and Suppresses Prostatic Pain and Hypersensitivity in a Capsaicin Induced Prostatitis Model in Rats. Int. J. Mol. Sci..

[74] Juan Y.S., Chuang S.M., Long C.Y., Chen C.H., Levin R.M., Liu K.M., Huang C.H. (2012). Neuroprotection of green tea catechins on surgical menopause-induced overactive bladder in a rat model. Menopause.

[75] Juan Y.S., Chuang S.M., Lee Y.L., Long C.Y., Wu T.H., Chang W.C., Levin R.M., Liu K.M., Huang C.H. (2012). Green tea catechins decrease oxidative stress in surgical menopause-induced overactive bladder in a rat model. BJU Int..

[76] Resnick N.M., Yalla S.V. (1987). Detrusor hyperactivity with impaired contractile function. An unrecognized but common cause of incontinence in elderly patients. JAMA.

[77] Hu Z., Yang Y., Gao K., Rudd J.A., Fang M. (2016). Ovarian hormones ameliorate memory impairment, cholinergic deficit, neuronal apoptosis and astrogliosis in a rat model of Alzheimer’s disease. Exp. Ther. Med..

[78] Si D., Li J., Liu J., Wang X., Wei Z., Tian Q., Wang H., Liu G. (2014). Progesterone protects blood-brain barrier function and improves neurological outcome following traumatic brain injury in rats. Exp. Ther. Med..

[79] Vest R.S., Pike C.J. (2013). Gender, sex steroid hormones, and Alzheimer’s disease. Horm. Behav..

[80] Yoshiyama M., Mochizuki T., Nakagomi H., Miyamoto T., Kira S., Mizumachi R., Sokabe T., Takayama Y., Tominaga M., Takeda M. (2015). Functional roles of TRPV1 and TRPV4 in control of lower urinary tract activity: Dual analysis of behavior and reflex during the micturition cycle. Am. J. Physiol. Ren. Physiol..

[81] Qureshi H.J., Hussain G., Jafary Z.A., Bashir M.U., Latif N., Riaz Z. (2010). Calcium status in premenopausal and postmenopausal women. J. Ayub Med. Coll. Abbottabad.

[82] Andersson K.E. (2016). Potential Future Pharmacological Treatment of Bladder Dysfunction. Basic. Clin. Pharmacol. Toxicol..

[83] Caudle R.M., Karai L., Mena N., Cooper B.Y., Mannes A.J., Perez F.M., Iadarola M.J., Olah Z. (2003). Resiniferatoxin-induced loss of plasma membrane in vanilloid receptor expressing cells. Neurotoxicology.

[84] Wu Y., Qi J., Wu C., Rong W. (2021). Emerging roles of the TRPV4 channel in bladder physiology and dysfunction. J. Physiol..

[85] Grundy L., Daly D.M., Chapple C., Grundy D., Chess-Williams R. (2018). TRPV1 enhances the afferent response to P2X receptor activation in the mouse urinary bladder. Sci. Rep..

[86] Yoshizumi M., Tazawa N., Watanabe C., Mizoguchi H. (2022). TRPV4 activation prevents lipopolysaccharide-induced painful bladder hypersensitivity in rats by regulating immune pathways. Front. Immunol..

[87] Seki S., Sasaki K., Fraser M.O., Igawa Y., Nishizawa O., Chancellor M.B., de Groat W.C., Yoshimura N. (2002). Immunoneutralization of nerve growth factor in lumbosacral spinal cord reduces bladder hyperreflexia in spinal cord injured rats. J. Urol..

[88] Avelino A., Cruz F. (2006). TRPV1 (vanilloid receptor) in the urinary tract: Expression, function and clinical applications. Naunyn Schmiedebergs Arch. Pharmacol..

[89] Herrera G.M., Heppner T.J., Nelson M.T. (2000). Regulation of urinary bladder smooth muscle contractions by ryanodine receptors and BK and SK channels. Am. J. Physiol. Regul. Integr. Comp. Physiol..

[90] Lee H., Koh B.H., Peri L.E., Corrigan R.D., Lee H.T., George N.E., Bhetwal B.P., Xie Y., Perrino B.A., Chai T.C. (2017). Premature contractions of the bladder are suppressed by interactions between TRPV4 and SK3 channels in murine detrusor PDGFRalpha(+) cells. Sci. Rep..

[91] Ghosh T.K., Eis P.S., Mullaney J.M., Ebert C.L., Gill D.L. (1988). Competitive, reversible, and potent antagonism of inositol 1,4,5-trisphosphate-activated calcium release by heparin. J. Biol. Chem..

[92] Kobayashi S., Kitazawa T., Somlyo A.V., Somlyo A.P. (1989). Cytosolic heparin inhibits muscarinic and alpha-adrenergic Ca2+ release in smooth muscle. Physiological role of inositol 1,4,5-trisphosphate in pharmacomechanical coupling. J. Biol. Chem..

[93] Climent B., Santiago E., Sanchez A., Munoz-Picos M., Perez-Vizcaino F., Garcia-Sacristan A., Rivera L., Prieto D. (2020). Metabolic syndrome inhibits store-operated Ca(2+) entry and calcium-induced calcium-release mechanism in coronary artery smooth muscle. Biochem. Pharmacol..

[94] Gevaert T., Vanstreels E., Daelemans D., Franken J., Van Der Aa F., Roskams T., De Ridder D. (2014). Identification of different phenotypes of interstitial cells in the upper and deep lamina propria of the human bladder dome. J. Urol..

[95] Koh B.H., Roy R., Hollywood M.A., Thornbury K.D., McHale N.G., Sergeant G.P., Hatton W.J., Ward S.M., Sanders K.M., Koh S.D. (2012). Platelet-derived growth factor receptor-alpha cells in mouse urinary bladder: A new class of interstitial cells. J. Cell Mol. Med..

[96] Lee H., Koh B.H., Peri L.E., Sanders K.M., Koh S.D. (2014). Purinergic inhibitory regulation of murine detrusor muscles mediated by PDGFRalpha+ interstitial cells. J. Physiol..

[97] Sanders K.M. (1996). A case for interstitial cells of Cajal as pacemakers and mediators of neurotransmission in the gastrointestinal tract. Gastroenterology.

[98] Foong D., Zhou J., Zarrouk A., Ho V., O’Connor M.D. (2020). Understanding the Biology of Human Interstitial Cells of Cajal in Gastrointestinal Motility. Int. J. Mol. Sci..

[99] Feng J., Gao J., Zhou S., Liu Y., Zhong Y., Shu Y., Meng M.S., Yan J., Sun D., Fang Q. (2017). Role of stem cell factor in the regulation of ICC proliferation and detrusor contraction in rats with an underactive bladder. Mol. Med. Rep..

[100] Levanovich P.E., Diokno A., Hasenau D.L., Lajiness M., Pruchnic R., Chancellor M.B. (2015). Intradetrusor injection of adult muscle-derived cells for the treatment of underactive bladder: Pilot study. Int. Urol. Nephrol..

[101] Zhang J., Kang N., Yu X., Ma Y., Pang X. (2017). Radial Extracorporeal Shock Wave Therapy Enhances the Proliferation and Differentiation of Neural Stem Cells by Notch, PI3K/AKT, and Wnt/beta-catenin Signaling. Sci. Rep..

[102] Weihs A.M., Fuchs C., Teuschl A.H., Hartinger J., Slezak P., Mittermayr R., Redl H., Junger W.G., Sitte H.H., Runzler D. (2014). Shock wave treatment enhances cell proliferation and improves wound healing by ATP release-coupled extracellular signal-regulated kinase (ERK) activation. J. Biol. Chem..

[103] Wang B., Zhou J., Banie L., Reed-Maldonado A.B., Ning H., Lu Z., Ruan Y., Zhou T., Wang H.S., Oh B.S. (2018). Low-intensity extracorporeal shock wave therapy promotes myogenesis through PERK/ATF4 pathway. Neurourol. Urodyn..

[104] Zhu G.Q., Jeon S.H., Bae W.J., Choi S.W., Jeong H.C., Kim K.S., Kim S.J., Cho H.J., Ha U.S., Hong S.H. (2018). Efficient Promotion of Autophagy and Angiogenesis Using Mesenchymal Stem Cell Therapy Enhanced by the Low-Energy Shock Waves in the Treatment of Erectile Dysfunction. Stem Cells Int..

[105] Seo M., Lim D., Kim S., Kim T., Kwon B.S., Nam K. (2021). Effect of Botulinum Toxin Injection and Extracorporeal Shock Wave Therapy on Nerve Regeneration in Rats with Experimentally Induced Sciatic Nerve Injury. Toxins.

[106] An J.Y., Yun H.S., Lee Y.P., Yang S.J., Shim J.O., Jeong J.H., Shin C.Y., Kim J.H., Kim D.S., Sohn U.D. (2002). The intracellular pathway of the acetylcholine-induced contraction in cat detrusor muscle cells. Br. J. Pharmacol..

